# Global alterations to the choroid plexus blood-CSF barrier in amyotrophic lateral sclerosis

**DOI:** 10.1186/s40478-020-00968-9

**Published:** 2020-06-26

**Authors:** J. Saul, E. Hutchins, R. Reiman, M. Saul, L. W. Ostrow, B. T. Harris, K. Van Keuren-Jensen, R. Bowser, N. Bakkar

**Affiliations:** 1 Department of Neurobiology, St. Joseph’s Hospital and Medical Center and Barrow Neurological Institute, 350 W Thomas Road, Phoenix, AZ 85013 USA; 2 Gregory W. Fulton ALS Center, Barrow Neurological Institute, Phoenix, AZ USA; 3 Neurogenomics Division, Translational Genomics Research Institute (Tgen), Phoenix, AZ USA; 4 Department of Biomedical Informatics, Arizona State University, Tempe, AZ USA; 5 Department of Neurology, Johns Hopkins University School of Medicine, Baltimore, MD USA; 6 Departments of Pathology and Neurology, Georgetown University Medical Center, Washington, DC USA

**Keywords:** Choroid plexus, ALS, Blood-CSF barrier, RNA sequencing, Tight junctions

## Abstract

The choroid plexus (CP) is a highly vascularized structure located in the ventricles that forms the blood-CSF barrier (BCSFB) and separates the blood from the cerebrospinal fluid (CSF). In addition to its role as a physical barrier, the CP functions in CSF secretion, transport of nutrients into the central nervous system (CNS) and a gated point of entry of circulating immune cells into the CNS. Aging and neurodegeneration have been reported to affect CP morphology and function and increase protein leakage from blood to the CSF. Amyotrophic lateral sclerosis (ALS) is a neurodegenerative disease associated with both upper and lower motor neuron loss, as well as altered proteomic and metabolomic signatures in the CSF. The role of the BCSFB and the CP in ALS is unknown. Here we describe a transcriptomic and ultrastructural analysis of BCSFB and CP alterations in human postmortem tissues from ALS and non-neurologic disease controls. ALS-CP exhibited widespread disruptions in tight junctional components of the CP epithelial layer and vascular integrity. In addition, we detected loss of pericytes around ALS blood vessels, accompanied by activation of platelet aggregation markers vWF and Fibrinogen, reminiscent of vascular injury. To investigate the immune component of ALS-CP, we conducted a comprehensive analysis of cytokines and chemokine panels in CP lysates and found a significant down-regulation of M-CSF and V-CAM1 in ALS, as well as up-regulation of VEGF-A protein. This phenotype was accompanied by an infiltration of MERTK positive macrophages into the parenchyma of the ALS-CP when compared to controls. Taken together, we demonstrate widespread structural and functional disruptions of the BCSFB in human ALS increasing our understanding of the disease pathology and identifying potential new targets for ALS therapeutic development.

## Introduction

The choroid plexus (CP) is a complex highly vascularized structure comprised of a tight polarized epithelial cell layer surrounding a stroma with highly fenestrated blood vessels and forms the blood-CSF barrier (BCSFB) [29, 40, 59]. The CP is located inside of the four ventricles and functions in the active transepithelial transport of nutrients into the central nervous system (CNS) and the removal of metabolic by-products out of the CNS. CP transcriptome analysis revealed that the choroid epithelium is a source of many growth factors that support the proliferation of ventricular-subventricular zone (V-SVZ) neural stem cells [67], and regulates the immune response in inflammatory conditions [35]. Unlike the blood brain barrier (BBB), the BCSFB is characterized by fenestrated capillaries, with the real “barrier” function being performed by the CP epithelium. Adjacent CP epithelial cells are bound by tight junctions that form the BCSFB, comprised of occludin, claudins 1–3 and 11 and zona occludens 1 (ZO-1). The CP is considered to be an active immunomodulatory gate, as opposed to a passive inert barrier [65], with M2 macrophages recruited to spinal cord injury sites entering through the CP and trafficking along the CSF to the injury site [66]. Healthy human CSF is devoid of neutrophils, and is instead populated by CD4+ memory T cells [41, 57] that enter the CSF through Selectin and integrin-mediated adhesion to the CP. CSF chemokine and leukocyte populations vary greatly under normal and pathological conditions [49], highlighting the dynamic interaction between the CP and the immune system, to support the continuous immunosurveillance and response to disease and injury.

CP morphology and function change with normal aging and neurodegenerative diseases such as Alzheimer’s disease (AD), including decreasing CSF production and turnover by as much as 50%, altering the levels of various proteins and enzymes involved in energy production, transport and free radical scavenging, but also increasing protein leakage from blood to the CSF [63, 64]. In particular, AD exhibits decreased CP epithelial cell height compared to age-matched controls [64], and the increased accumulation of Lipofuscin vacuoles and Biondi bodies [74]. In addition, increased Aβ deposition in the CP, potentially via uptake from the CSF, has been observed in AD [20, 73]. Increased deposition of Aβ oligomers in turn activates CP matrix metalloproteinases (MMPs) which decreases occludin, E-cadherin and claudin levels and thereby disrupt BCSF barrier integrity [11].

ALS is a fatal neurodegenerative disease that typically leads to death within 2–5 years of diagnosis. ~ 90% of the cases are considered sporadic, while 10% of ALS cases are familial, with pathogenic gene mutations in ~ 30 identified genes implicated in roughly 2/3rds of familial and 10% of sporadic cases [60, 79]. ALS is characterized by a progressive loss of upper and lower motor neurons, leading to paralysis and eventual death. The search of more effective therapies is hindered by the lack of understanding of mechanisms contributing to disease spread and motor neuron death. Potential mechanisms of disease spread include direct anatomical connectivity within the CNS [58] and recently proposed spread via the CSF [68]. Multiple studies have shown BBB and blood-spinal cord barrier (BSCB) impairments in human ALS as well as the mutant SOD1 model of disease [26]. For example, IgG and C3/C4 complement deposits in the spinal cord and motor cortex of ALS were first described by Donnenfeld and colleagues over 35 years ago [21]. Endothelial cell degeneration, capillary leakage, perivascular edema, tight junctional protein downregulation and microhemorrhages are other common signs of barrier damage reported in ALS patients as well as ALS mice models [25, 27, 32, 76]. Pericyte degeneration, perivascular basement membrane collagen IV expansion, and white matter capillary abnormalities are significant BBB-related pathologies in ALS patients that have not been detected in animal models of ALS.

Our group, and others, have reported altered levels of many proteins in CSF from ALS compared to controls, including inflammatory proteins, as well as cytoskeletal and extracellular matrix proteins, suggesting impaired CP barrier permeability [8, 17, 61, 77]. ALS patient CSF shows elevated total protein levels [30, 55], as well as increased CSF to serum ratios of albumin, IgG and complement C3 compared to non-neurological healthy controls [1, 2, 9, 44], indicating a leakage from the blood into the CSF and/or decreased clearance from the CSF. More recently, Schwartz et al. described an impaired activation of the CP in the G93A SOD1 ALS mouse model, possibly driven by decreased IFN-γ, leading to impaired recruitment of CD4+ T cells into the CP and the CSF [43]. In contrast to the mouse model, a recent study described significantly increased levels of IFN-γ in both CSF and serum of ALS patients [45], highlighting the inherent differences in disease mechanisms between human disease and SOD1 rodent models, and supporting the need to examine the CP in the human disease.

Given the central role of the CP in CSF maintenance and its function as an immune gatekeeper, we sought to investigate global transcriptomic and histopathological changes in post-mortem CP of ALS and non-neurologic disease controls. We discovered global disruption of cellular adhesion markers at the protein and mRNA levels in ALS-CP, as well as vascular and pericyte disruptions. In addition, ALS-CP exhibited marked activation and aggregation of platelets and striking macrophage infiltration into the CP stroma, accompanied by increased VEGF-A protein and decreased metalloproteinase levels. Taken together, our findings highlight disruption of the BCSFB structure and function as a novel aspect of ALS pathogenesis.

## Results

### Global changes in CP ALS transcriptome

**Fig. 1:**
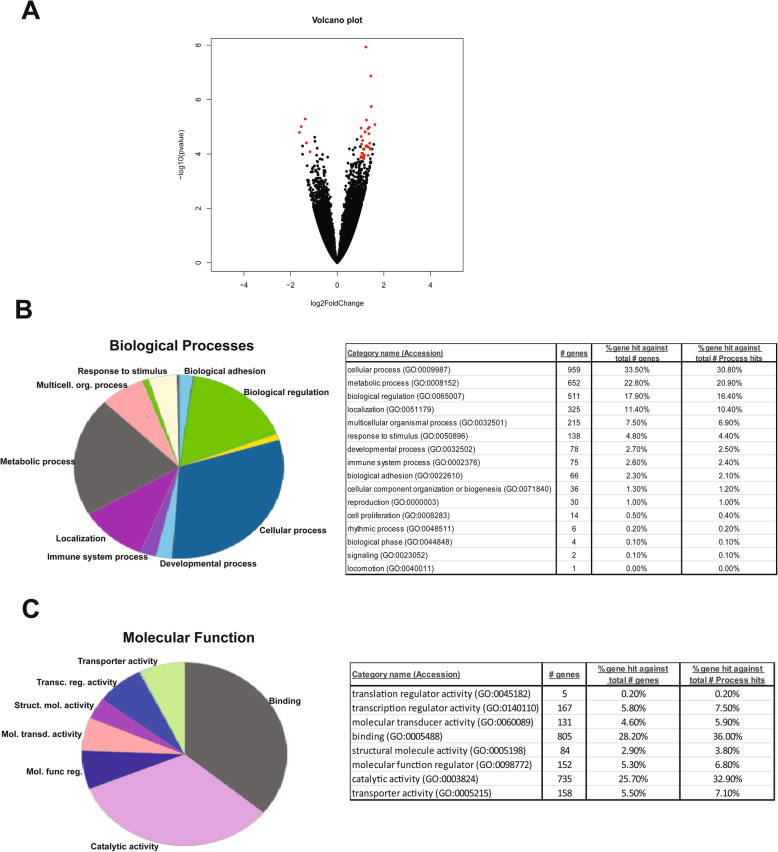
**a** Volcano plot of significantly altered genes from the RNA-seq analysis on *n* = 11 non-neurological disease controls, *n* = 14 ALS cases (sporadic and C9-ALS). The primary X axis shows the log2 (fold change) of controls vs ALS, while the secondary axis designates the −log10 (*p* value), analyzed with the following parameters (adjusted *p*-values (*p* < 0.05), a fold change greater than 2, and a baseMean value greater than 20). The red dots mark data points that are significantly altered in either direction. Relaxed analysis parameters were used for pathway analysis (adjusted *p*-values (*p* < 0.1), a fold change greater than 1.5, and a baseMean value greater than 20) and pie graphs and tables visualizing the gene ontology (GO) distribution of genes detected in both control and ALS groups classified by using Panther terms for biological processes **b** and molecular functions **c**

**Fig. 2:**
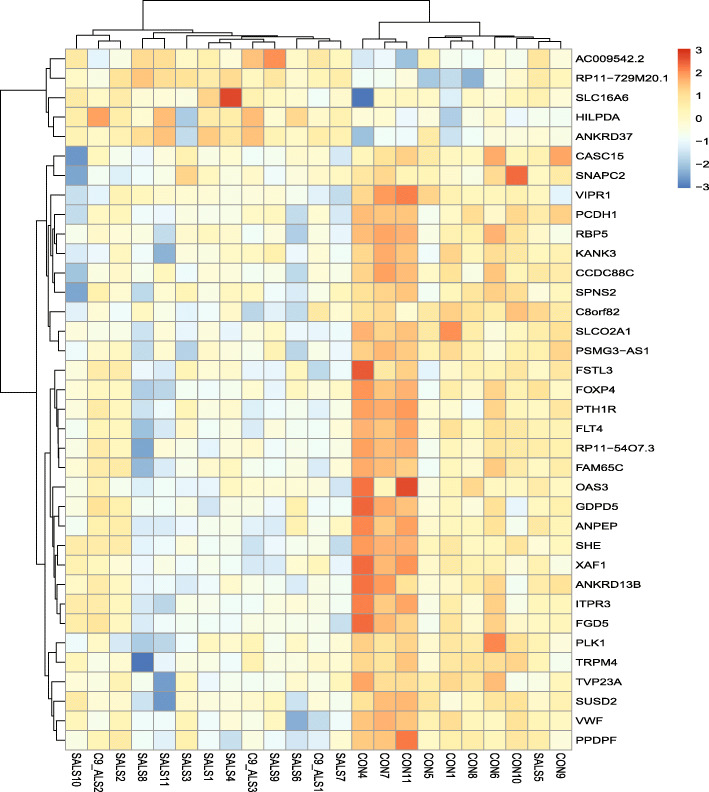
Heatmap of the Z-transformed regularized log gene expression values of top significantly altered genes, which highlights differences between control and ALS (sporadic and C9-ALS). Upregulated genes are red, while downregulated genes are blue. Sample names are shown on the bottom (CON vs SALS vs C9-ALS) while gene names are on the right. Case demographics can be found in the demographics table in the supplemental materials

To evaluate the ALS-CP transcriptome, we performed RNA sequencing (RNAseq) of the right lateral ventricles of non-neurologic disease controls (*n* = 11), sporadic ALS cases (*n* = 11) and C9-ALS (*n* = 3). Age did not differ between the control (average 65.3 + 12.7 years) and the ALS groups (C9-ALS + SALS; average 66.6 + 10.8). Two controls were statistically dropped out of the analysis due to their clustering discrepancy in PCA plots. The C9-ALS group clustered with the SALS and so the two groups were merged into one common ALS group. Overall, there were 48 differentially expressed genes (fold change > 2; *p*-adjusted < 0.05; average across all samples> 20), with the majority of transcripts being downregulated in ALS choroid plexus and only a minority of transcripts upregulated, a phenotype previously described in CP of subjects with major depressive disorder ([72] volcano plot, Fig. 1a and heatmap Fig. 2). Among differentially regulated genes were solute carrier transmembrane transporters (SLC16A6 and SLCO2A1) and cytoskeletal proteins protocadherin 1 (PCDH1) and KANK3 (KN Motif And Ankyrin Repeat Domains 3). Other differentially regulated genes were VEGF receptor (Fms-related tyrosine kinase 4) Flt4, pericyte marker CD13/ANPEP and platelet activation marker (Von Willebrand factor VWF).

We used ToppGene Suite to identify pathways enriched in an enlarged dataset generated through relaxed analysis parameters ([14]; Table 1). Results showed top pathways enriched in ALS choroid plexus to be “NOTCH signaling”, “endothelin signaling”, “platelet activation”, and “integrin signaling”. All detected CP proteins were also classified using Gene Ontology (GO) annotation. Figure 1b-c shows the distribution of the identified proteins among the GO-terms within the groups: molecular function, and biological process, using Panther. For molecular function gene ontology, the proteins were predominantly enzymes, molecule binding proteins, structural molecules, and transporters. Biological processes altered in ALS-CP pertained to metabolic processes, localization, biological adhesion and immune regulation.

**Table 1:** ToppGene Pathways enriched in ALS choroid plexus

Pathway ID	Name	***p***-value	q-value Bonferroni	Hit Count in List	Hit Count in Genome	Hit in Query List
1,269,530	Signaling by NOTCH	2.38E-06	7.21E-03	35	113	ADAM10,ARRB1,ARRB2,HDAC3,HDAC9,HDAC4,MAML1,ITCH,HDAC5,MAMLD1,HEYL,EP300,FCER2,ST3GAL6,HDAC7,TMED2,LFNG,MFNG,MOV10,NOTCH1,NOTCH2,NOTCH3,NOTCH4,FURIN,NEURL1B,MAML3,SKP1,PSENEN,NCSTN,HEY1,TLE1,TLE2,TLE3,HEY2,TP53
P00019	Endothelin signaling pathway	9.20E-06	2.79E-02	25	73	ADCY2,ADCY3,ADCY5,ADCY7,ADCY4,PIK3R3,SEC11C,ECE1,EDN2,GNA11,GNAL,ITPR1,ITPR3,FURIN,PIK3C2A,PIK3C2B,PIK3C3,PIK3CD,PLCB2,PLCB3,PRKAR1A,PRKAR2B,PRKCE,PRKCI,SEC11A
PW:0000204	Notch signaling	2.85E-05	8.64E-02	15	35	ADAM10,NEURL2,MAML1,HEYL,LFNG,MFNG,NOTCH1,NOTCH2,NOTCH3,NOTCH4,MAML3,PSENEN,NCSTN,HEY1,HEY2
1,269,535	Signaling by NOTCH1	3.86E-05	1.17E-01	24	74	ADAM10,ARRB1,ARRB2,HDAC3,HDAC9,HDAC4,MAML1,ITCH,HDAC5,MAMLD1,HEYL,EP300,HDAC7,NOTCH1,NEURL1B,MAML3,SKP1,PSENEN,NCSTN,HEY1,TLE1,TLE2,TLE3,HEY2
952,858	Platelet activation	4.82E-05	1.46E-01	34	123	ADCY2,ADCY3,ADCY5,ADCY7,ADCY4,PIK3R3,PLA2G4C,ARHGEF1,COL1A1,COL1A2,COL3A1,RASGRP2,FCGR2A,GNA13,GNAI3,GUCY1B1,ITPR1,ITPR3,MYLK,PIK3CD,PLCB2,PLCB3,PLCG2,APBB1IP,PRKCI,MAPK11,MAPK13,RAP1B,MAPK12,SRC,TBXA2R,TLN1,VWF,P2RY12
P00034	Integrin signaling pathway	7.63E-05	2.31E-01	42	167	PIK3R3,ITGA10,ITGA8,RHOB,ARL1,CDC42,COL1A1,COL1A2,COL3A1,COL5A1,COL6A1,COL6A2,COL6A3,COL7A1,COL8A1,COL9A3,COL11A1,COL15A1,COL16A1,ELMO1,DNAJC27,LAMC3,PTK2B,ITGA3,ITGA9,LAMA2,MAP3K3,PIK3C2A,PIK3C2B,PIK3C3,PIK3CD,MAPK6,MAPK13,PXN,RAP1B,ITGA11,SHC1,LIMS2,PARVA,SRC,TLN1,COL14A1
102,279	Endocytosis	7.97E-05	2.42E-01	59	260	CLTCL1,ADRB1,GRK2,AP2A1,AP2A2,PIP5K1B,AMPH,ARRB1,ARRB2,RAB11A,CAPZA1,CAPZA2,CBL,USP8,CDC42,RAB11B,CYTH2,CYTH1,CLTB,VPS26A,RAB11FIP3,ACAP1,ZFYVE16,CHMP7,ARFGAP3,DNM2,ITCH,WASHC3,ERBB4,FGFR3,ACAP3,VTA1,ARFGAP2,CYTH4,EHD1,GRK5,SNF8,HLA-F,HSPA1A,HSPA8,KIF5B,CHMP1A,WIPF3,PLD2,PML,SH3KBP1,PRKCI,RAB4A,EHD3,EHD2,SH3GL3,VPS35,SRC,RBSN,TGFB1,ARAP3,PARD3,CBLC,WIPF1
908,257	Adrenergic signaling in cardiomyocytes	1.24E-04	3.77E-01	37	144	ADCY2,ADCY3,ADCY5,ADCY7,ADRA1D,ADRB1,ADCY4,ATP1B1,ATP2B4,CACNA1C,CACNA2D1,CACNB3,CACNB4,CALM1,CAMK2B,CREB3L4,RPS6KA5,CACNA2D2,ATF2,ATF6B,CREM,RAPGEF3,GNAI3,KCNE1,MYH6,MYL3,PLCB2,PLCB3,PPP2R3A,PPP2R5C,MAPK11,MAPK13,MAPK12,SCN1B,SCN4B,CACNA2D3,TPM2
1,269,537	NOTCH1 Intracellular Domain Regulates Transcription	1.52E-04	4.62E-01	17	48	HDAC3,HDAC9,HDAC4,MAML1,HDAC5,MAMLD1,HEYL,EP300,HDAC7,NOTCH1,MAML3,SKP1,HEY1,TLE1,TLE2,TLE3,HEY2
121,494	Dilated cardiomyopathy	1.66E-04	5.03E-01	26	90	ADCY2,ADCY3,ADCY5,ADCY7,ADRB1,ADCY4,ITGA10,ITGA8,CACNA1C,CACNA2D1,CACNB3,CACNB4,CACNA2D2,DES,ITGA3,ITGA9,LAMA2,MYH6,MYL3,ITGA11,SGCA,SGCB,SGCG,CACNA2D3,TGFB1,TPM2

A further analysis of biological processes was then performed using multiple gene ontology analyses including DAVID and ToppGene to avoid background annotation bias and focus on commonalities (Table 2, and Supplemental Table 1). Common top biological processes altered in ALS-CP pertained to cellular adhesion, vasculature/blood vessel development/VEGF signaling, and platelet activation. It is noteworthy that some biological processes that were unique to the DAVID GO analysis pertained to neuron projection and apoptosis, acetylcholine receptor signaling, and chemotaxis potentially highlighting the role of the CP in regulation of neuronal growth factors.

**Table 2:** Analysis of GO Biological processes using DAVID

GO Biological Processes	Count	%	***P***Value	Fold Enrichment
GO:0070208 ~ protein heterotrimerization	9	0.308325	1.54E-04	4.755443675
GO:0001501 ~ skeletal system development	35	1.199041	2.61E-04	1.889835686
GO:0007219 ~ Notch signaling pathway	30	1.027749	5.39E-04	1.92974526
GO:0007155 ~ cell adhesion	88	3.014731	5.77E-04	1.418229632
GO:0007507 ~ heart development	42	1.438849	6.58E-04	1.697754026
GO:0048010 ~ vascular endothelial growth factor receptor signaling pathway	21	0.719424	0.001052	2.157562408
GO:0061337 ~ cardiac conduction	15	0.513875	0.001791	2.465785609
GO:0007213 ~ G-protein coupled acetylcholine receptor signaling pathway	8	0.274066	0.00194	3.945256975
GO:0030168 ~ platelet activation	28	0.959233	0.002548	1.801095576
GO:0050921 ~ positive regulation of chemotaxis	7	0.239808	0.002691	4.315124816
GO:0010976 ~ positive regulation of neuron projection development	23	0.787941	0.003133	1.911676484
GO:0035556 ~ intracellular signal transduction	75	2.569373	0.003355	1.37667931
GO:0043525 ~ positive regulation of neuron apoptotic process	14	0.479616	0.00342	2.408441758
GO:0007569 ~ cell aging	10	0.342583	0.003925	2.958942731
GO:0048844 ~ artery morphogenesis	9	0.308325	0.004344	3.170295784
GO:0045333 ~ cellular respiration	9	0.308325	0.004344	3.170295784
GO:0033138 ~ positive regulation of peptidyl-serine phosphorylation	19	0.650908	0.004583	2.007853996
GO:0043547 ~ positive regulation of GTPase activity	99	3.391572	0.005103	1.296174028
GO:0003184 ~ pulmonary valve morphogenesis	6	0.20555	0.006259	4.438414097
GO:0003151 ~ outflow tract morphogenesis	14	0.479616	0.006454	2.251369469

### ALS alters BCSFB integrity

**Fig. 3:**
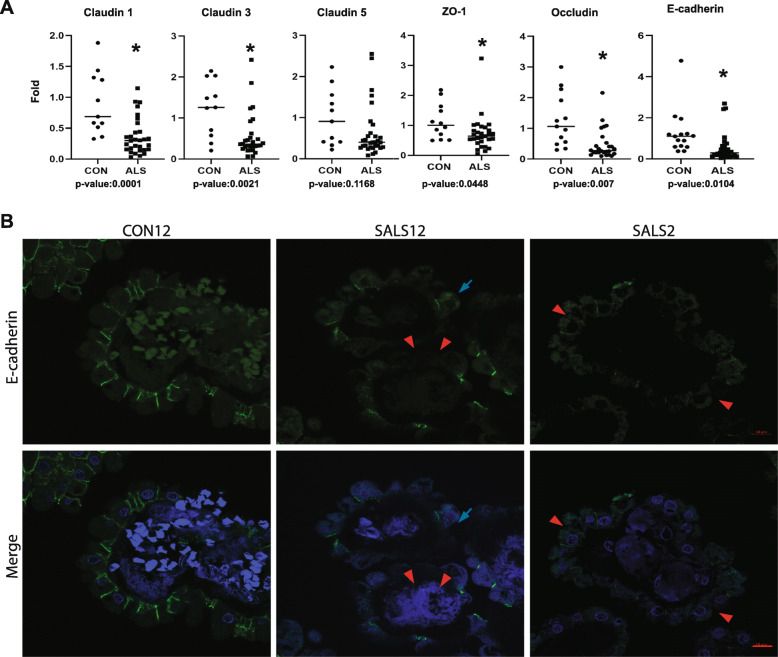
**a** RNA was extracted and quantitative real-time PCR analysis was performed on *n* = 15 controls and *n* = 31 SALS-CP tissues looking for changes in tight junction and adhesion markers previously implicated in CP function/disease. Asterisks denote significance using Student t-test and individual *p*-values are shown under the respective graphs. **b** Immunofluorescence for E-cadherin was performed on *n* = 3 controls and *n* = 5 ALS cases. Note the tight intercellular staining in controls, compared to missing or decreased staining from some CP cells (red arrowheads), or diffuse cytoplasmic stain (blue arrows). DAPI was used to counterstain nuclei

Given that cellular adhesion was among the top altered biological processes in ALS-CP, and given the crucial role for this layer as a barrier, we validated and further explored tight junction and adhesion factors expressed in the CP. We first performed a Q-PCR validation of specific adhesion markers in a larger cohort of non-neurologic disease controls and ALS-CP samples (Fig. 3a). We examined Claudin 1, Claudin 3, Claudin 5, Occludin and ZO-1 in 31 ALS-CP samples compared to 15 non-neurologic disease controls. While Claudin 1, Claudin 3, Occludin, and E-cadherin were significantly downregulated (*p*-values of 0.0001, 0.0021, 0.007, and 0.0104, respectively), ZO-1 also reached significance (*p*-value of 0.0448), and Claudin 5 was not significantly altered (*p*-value of 0.1168). Interestingly, Claudin 5 transcript has recently been shown to be significantly downregulated in FTD but not Huntington’s or Alzheimer’s disease choroid plexus [70].

To define the localization of adhesion markers in ALS-CP, we focused on E-cadherin (Fig. 3b). In control CP, E-cadherin specifically marks intercellular choroidal junctions. In ALS-CP however, we observed a mislocalization of this marker, with many cases showing basal or diffuse cytoplasmic localization of E-cadherin (blue arrows), while other cases show an overall absence from intercellular junctions (red arrowheads). ZO-1 immunofluorescence showed a similar pattern of overall loss in some ALS samples (Fig. S1, middle panel), while other samples were characterized by regional losses of the adhesion marker (red arrowheads, Fig. S1). Additional adhesion markers examined, Claudin 5 showed regional losses from endothelial cells in ALS CP (Fig. S2A, black arrows). Additionally, we observed Claudin 5 staining in nucleated cells located within blood vessel lumen in most ALS cases (Fig. S2A, red arrowheads), although the nature of these cells requires further investigations. Consistent with the lack of significant changes in Claudin 5 mRNA, protein levels of this adhesion marker were not significantly altered in ALS by western blot analysis (Fig. S2B). Both Claudin 3 and Occludin protein levels exhibited large variations within ALS samples, with some showing down-regulation of the two markers (in samples grouped as ALS cohort 1, Fig. S2B), while others were characterized by no changes or even increases (samples grouped as cohort 2, Fig. S2B). Taken together, these findings support a global disruption of the BCSFB integrity in ALS-CP.

### ALS activates platelet aggregation pathways

**Fig. 4:**
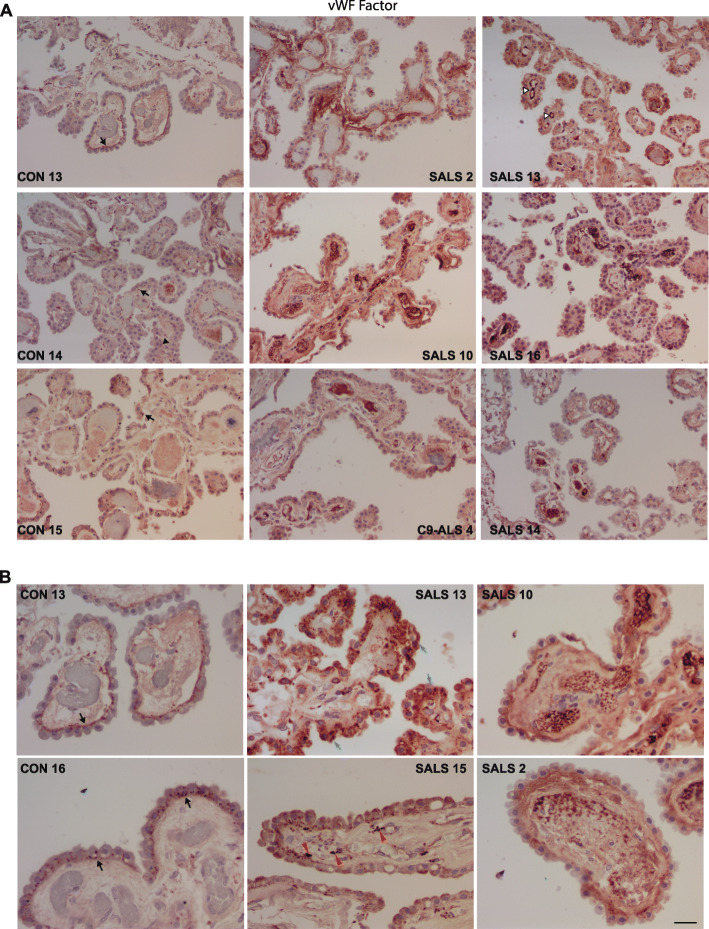
**a** and **b** Immunohistochemistry for vWF was performed on *n* = 5 controls and *n* = 7 ALS cases. Pictures were taken under low magnification to show the spread of positive staining throughout the tissue **a** and at 40x magnification **b** to show localization in the stroma (red arrowheads), underneath the basal side of the choroid plexus epithelial layer (black arrows), and within CP cells (blue arrows). White arrowheads point to staining within endothelial cells, while black arrowheads point to interstitial staining in some controls. Scale bar: 20 μm

Since platelet activation was significantly altered in ALS-CP by pathway analysis of the transcriptomic data, we next examined markers of platelet activation in CP tissue. Platelets play a key role in hemostasis at sites of vascular injury where they rapidly bind damaged blood vessel walls and aggregate to form a thrombus, thus preventing excessive bleeding [78]. Platelets also bind to sites of endothelial cell erosion, stimulating thrombus formation and promoting atherothrombotic disease [37]. Platelet adhesion to the extracellular matrix, followed by release of the highly procoagulatory glycoprotein von Willebrand factor (vWF) is the first step in the initiation of the coagulation cascade. vWF is stored in endothelial cell Weibel–Palade bodies (WPB), in α-granules of platelets, as well as in subendothelial connective tissue [54]. vWF in control CP showed a punctate staining pattern typically associated with endothelial staining on the basal side of CP epithelial cells, and virtually no or little vWF within blood vessel lumen (Fig. 4a and b, controls). Some controls exhibited interstitial vWF punctate staining (2/6 controls, black arrowhead). Little vWF was evident in CP endothelial cells, with most staining located to the basal side of CP epithelial cells. By contrast, in ALS-CP, large vWF aggregates filled the lumen of blood vessels and could be seen on the surface of platelets inside blood vessels (Fig. 4a and b), as well as inside endothelial cells (white arrowheads, Fig. 4a). We observed clearly visible ultra-large vWF fibers (defined as vWF fibers ≥5 μm) typically associated with platelet adhesion and aggregation in many ALS-CP vessels. Darker vWF puncta, as well as larger aggregates were also observed in the stroma surrounding blood vessels (red arrowheads, Fig. 4b), as well as inside the CP epithelial cells (blue arrows, Fig. 4b).

**Fig. 5:**
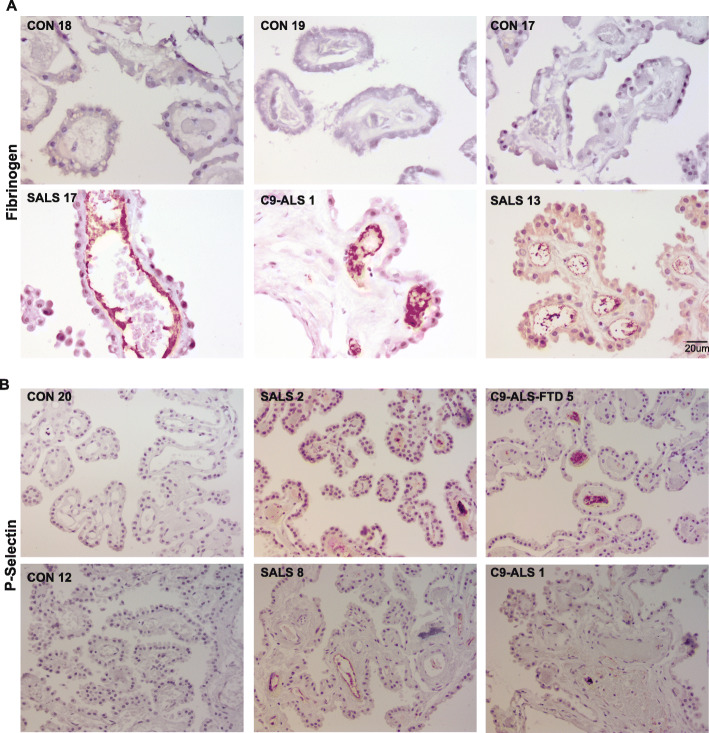
**a** Immunohistochemical analysis of Fibrinogen staining in ALS (SALS and C9-AL) and controls. Pictures were taken at 40x magnification. Scale bar: 20 μm. **b** Staining for P-selectin (CD62P) was performed in *n* = 4 control CP tissues, *n* = 10 SALS and *n* = 5 C9-ALS/C9-ALS-FTD cases

**Fig. 6:**
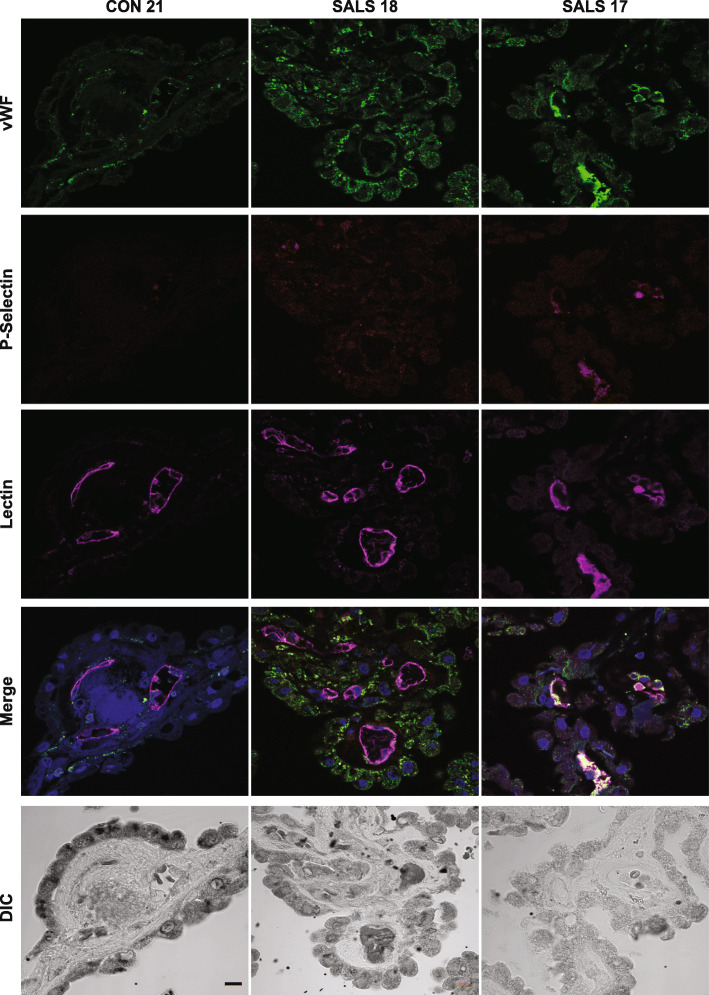
Immunofluorescence for vWF and P-selectin was performed on n=6 ALS and n=3 control CP tissues and imaged under 63x using confocal microscopy. Lectin was used to mark blood vessels and DAPI denotes nuclei. DIC (differential interference contrast) images were taken to show tissue morphology. Scale bar: 10um

Aside from vWF, platelet α-granules also contain adhesive glycoproteins such as P-selectin (CD62P) and fibrinogen as well as angiogenic factors, fibrinolytic inhibitors, and various chemokines [78]. These factors play an important role in hemostasis but are also involved in modulating the immune response by releasing cytokines that affect leukocyte function [36]. Fibrinogen cross-links platelets and contributes to thrombus stabilization, while P-selectin mediates the interactions between platelets, leukocytes, and endothelial cells, thereby regulating the release of proinflammatory cytokines. We investigated the levels of fibrinogen and P-selectin in control and ALS-CP tissues. While Fibrinogen was undetectable in control CP, ALS CP demonstrated dark positive staining on endothelial surfaces, inside blood vessel lumens, and on the surface of platelets (Fig. 5a). A similar staining pattern was observed for P-selectin, with both endothelial and luminal vessels positive for this protein in ALS but not control CP (Fig. 5b). To show that vWF aggregates were associated with platelet adhesion /aggregation, we co-immunolocalized vWF with P-selectin in control and ALS-CP (Fig. 6, *n* = 3 controls, *n* = 6 ALS). We show again that vWF is low in controls and limited to the basal side of CP cells, while P-selectin could not be detected. ALS-CP showed dramatic increases in overall vWF levels in the stroma and sub-choroidal compartment as noted in Fig. 4 (Fig. 6, middle panel). Interestingly, lumenal vWF ultra-large aggregates were positive for P-selectin.

Taken together, these data support an activation of platelet aggregation pathways, along with release of thrombus-forming factors, presumably resulting from vascular damage.

### Vascular integrity is compromised in ALS-CP

**Fig. 7:**
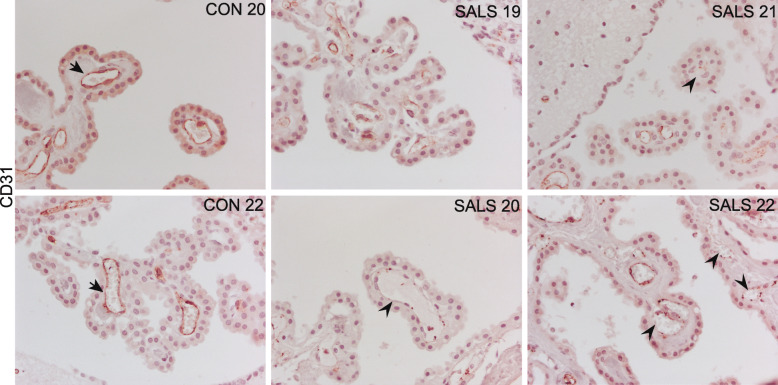
Immunohistological characterization of CD31 staining in *n* = 10 ALS and *n* = 6 control CP tissue samples. Black arrows point to continuous staining on the surface of endothelial cells, while black arrowheads highlight areas of discontinuous staining

In order to further investigate the potential vascular damage in ALS-CP, we focused on CD31, also known as platelet endothelial cell adhesion molecule-1 (PECAM-1), an endothelial cell marker that localizes to intercellular junctions, and plays an important role in endothelial cell adhesion, but also implicated in T-cell homeostasis and trafficking [46, 48, 56]. In control CP tissue, CD31 strongly labels endothelial cells with no staining in the stroma or CP layer (arrows, Fig. 7a, *n* = 6). ALS-CP on the other hand, exhibits global decreased levels of CD31, but also a discontinuous punctate staining pattern in some regions (Fig. 7a, arrowheads). It is unclear if this decrease is specific for the CD31 protein or underlies an actual loss of endothelial cells.

**Fig. 8:**
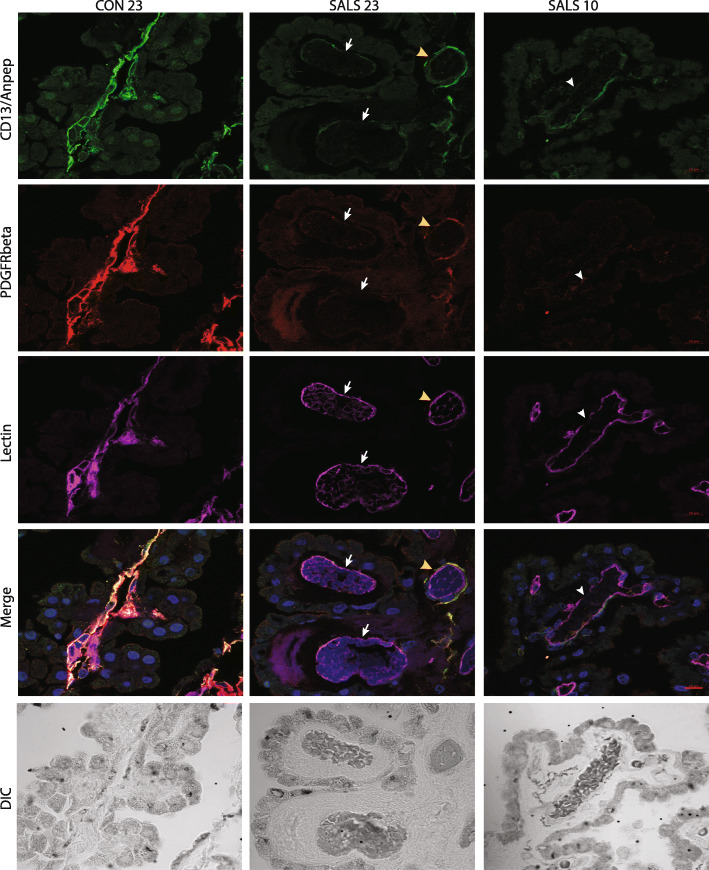
Immunofluorescence staining for pericyte markers CD13/ANPEP and PDGFR-β was performed on control and ALS-CP tissues. Slides were imaged under 63x using confocal microscopy. Lectin was used to mark blood vessel and DAPI stains nuclei. White arrows point to blood vessel with no staining for either pericyte marker, while white arrowheads show loss of one of the marker. Yellow arrows show ALS blood vessels with spared pericytes. Scale bar: 10um

In an effort to link CD31 loss to vascular function, we next focused on pericytes, “contractile” cells abundant in the brain that modulate brain vessel permeability as part of the neurovascular unit [3]. Since the CD13/ANPEP pericyte marker transcript was significantly downregulated in the ALS-CP RNA-seq analysis (Fig. 2), we focused on ANPEP as well as another pericyte marker PDGFRβ in ALS-CP compared to controls. Both pericyte markers were detected in control CP, co-localized in the same cells and surrounding blood vessel basement membrane marked with lectin (Fig. 8 and S3B, black arrows). In ALS, varying degrees of pericyte loss were observed and overall CD13/ANPEP signal was significantly downregulated (Fig. S3A and B red arrowheads). PDGFRβ signal failed to reach significance potentially due to high variability within the control group (Fig. S3A). Some blood vessels retained expression of both overlapping markers (Fig. 8, middle panel, yellow arrowhead), while other blood vessels had severe loss of both pericyte markers CD13/ANPEP and PDGFRβ (white arrows, middle panel). We also observed loss of PDGFRβ but not CD13/ANPEP in some CP regions (Fig. 8, white arrowheads, right panel). Taken together, these findings support a loss of pericytes, potentially resulting in increased endothelial permeability, which coupled with the severe loss of the CD31 endothelial marker highlights a deregulation of BCSFB vascular integrity in ALS.

### Immune activation in ALS-CP

**Fig. 9:**
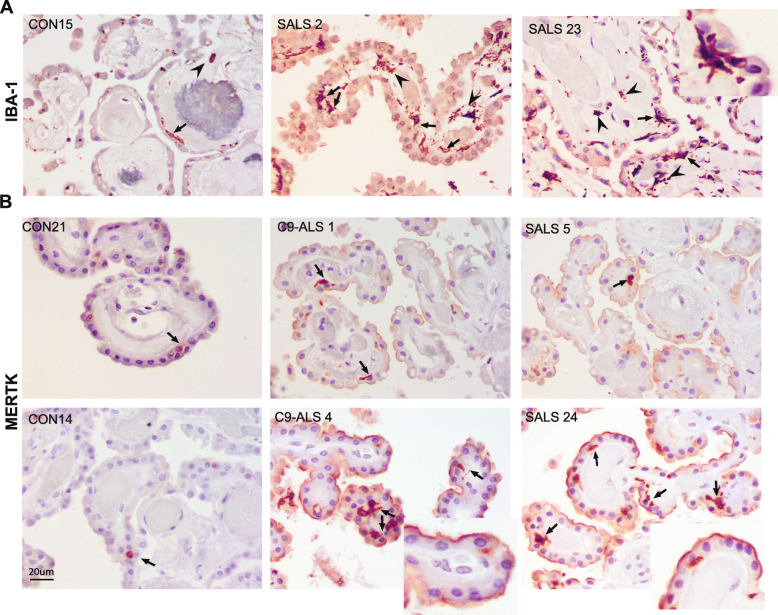
Immunohistochemical analysis for macrophage markers Iba-1 **a** and MERTK **b** in control and ALS-CP postmortem tissues. Arrows in **a** and **b** point to macrophage localization at the base of the CP epithelial cells, while arrowheads in **a** point to stromal localization.Insert in a shows a highly branched morphology of an Iba1-positive cell. Arrowheads in **b** point to apical MERTK staining on the surface of CP epithelial cells, while inserts in **b** highlight staining on the apical surface of CP cells. Scale bars: 20 μm

Since the CP contains resident immune cells including T cells and macrophages, we asked whether ALS alters the immune cell composition of the CP. We investigated the levels and localization of the macrophage markers Iba1 and MER Proto-Oncogene, Tyrosine Kinase (MERTK) in ALS-CP and non-neurological disease controls. In control CP, sparse Iba1 positive cells were located in the choroidal stroma (arrowhead), and in close proximity to the basal side of choroidal epithelia (arrows, Fig. 9a). A marked increase in the number of Iba1 positive macrophages was observed in ALS cases in the stroma as well as at the base of choroidal epithelial cells, with a highly branched morphology (insert). It is noteworthy that we rarely saw CD3 positive regulatory T cells (T reg) in ALS choroid plexus (Supplementary Fig S4A).

MERTK expression is characteristic of immunosuppressive M2-like macrophages and contributes to clearance of apoptotic cells as well as platelet activation [13, 16]. In control CP, MERTK positive cells are located in the choroid stroma, in close proximity to the basal side of the choroidal epithelial cells (Fig. 9b, arrows and Fig. S4B), similar to the Iba1 positive cells. The number of these MERTK positive macrophages dramatically increases in ALS-CP, with most cells similarly located on the stromal side of the epithelial cells (Fig. 9b, arrows and supplemental Fig. S4B for lower magnification). It is notable that we also observed MERTK expression on the apical surface of some choroidal cells in most ALS cases examined (Fig. 9b, inserts), although the significance of this expression within the CP epithelial cells is unknown. In addition to dendritic cells and macrophages, MERTK can be expressed by epithelial and endothelial cells [6], where it can drive efferocytosis thus promoting wound healing as well as down-regulation of pro-inflammatory signals.

**Fig. 10:**
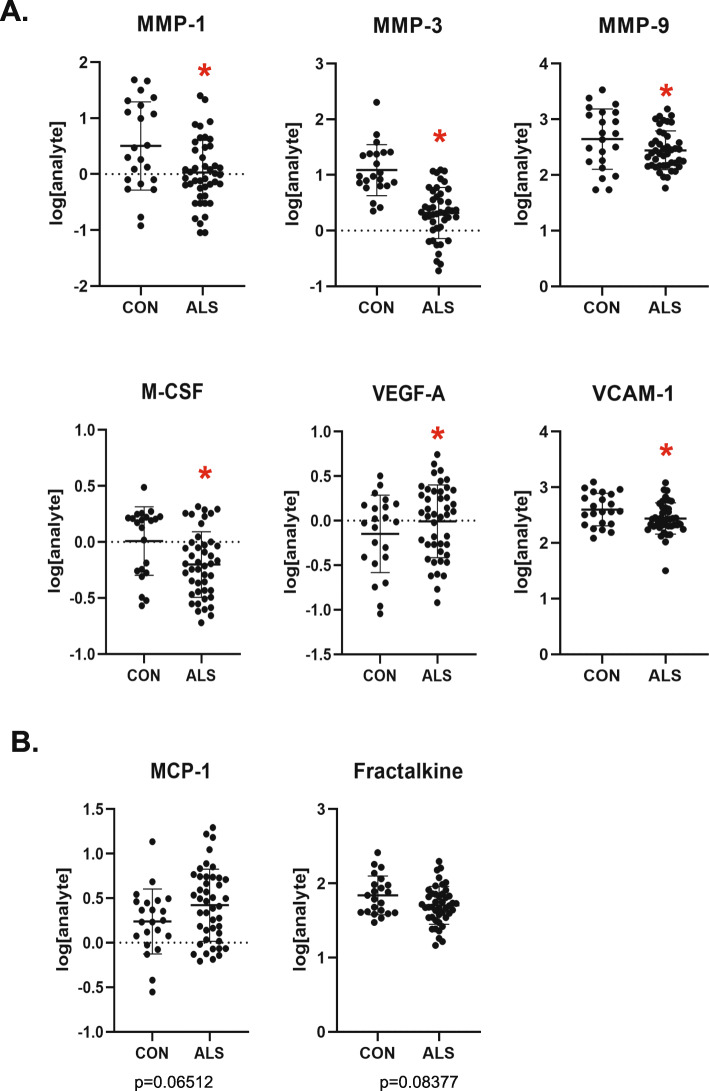
**a** Protein levels of various chemokines and metalloproteinases were measured by MesoScale Discovery assays, and results for *n* = 44 ALS and *n* = 22 controls CP samples were log-transformed and plotted. *p*-values were calculated through a logistic regression model that accounts for patient age (under 65 vs above 65), as well as the plate on which the sample was run to avoid batch effect bias. Graphs shown are for analytes with significant *p*-values≤0.05 (also denoted by red asterisks). **b** Analysis performed similar to **a** and analytes represented had *p*-values close to significance (exact values depicted on the figure). Raw data can be found in table S2

To further define the immune signature in the ALS-CP, we performed immunoassays on CP tissue lysates, focusing on chemokines and cytokines that have previously been shown to be altered in ALS patients and the SOD1G93A mouse model [43], as well as matrix metalloproteinases that have previously been implicated in Alzheimer’s disease BCSFB breakdown [10, 11]. We used 44 ALS samples (including *n* = 7 C9-ALS and *n* = 36 SALS) and 22 non-neurologic disease controls (Supplemental Table 2). Global results showed a large variability within the ALS and the control group, with a subset of each displaying an “inflammatory signature” (Fig. S5). No correlations of the immune signature could be drawn with patient gender, age, disease site of onset or disease progression rates. Here again, and similar to our RNA-seq transcriptomic analysis, CP samples harboring the *c9orf72* repeat expansion did not differ from SALS-CP samples (Figs. S5 and S6). Out of the analytes examined, 6 showed significant changes between the two groups (Figs. 10a and S5), and two additional trended toward significance (Fracktalkine/CX3CL1 and VCAM-1; Fig. 10b). All three examined metalloproteinases (MMP1, MMP3 and MMP9) were surprisingly downregulated in ALS compared to controls. M-CSF and V-CAM1 were also downregulated in ALS compared to controls, while VEGF-A was significantly increased in the ALS group (Fig. 10).

**Fig. 11:**
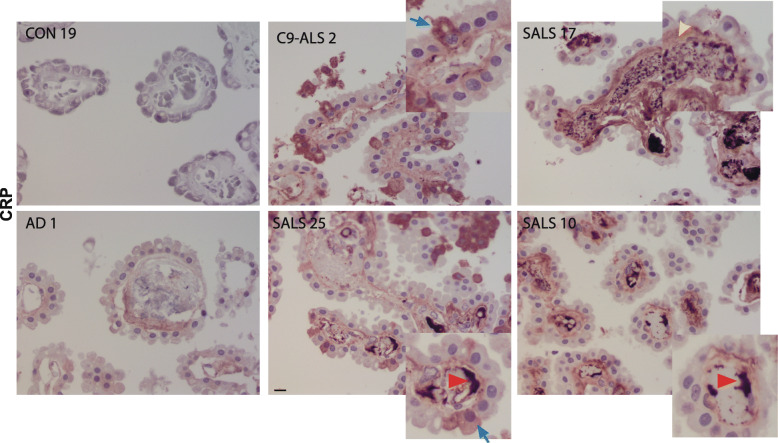
CRP immunohistochemical analysis of ALS and CON CP cases. Red arrowheads point to CRP deposition on luminal side of blood vessels, blue arrows point to CRP inside select CP epithelial cells in ALS, while yellow arrowheads point to stromal CRP staining. Scale bar: 20 μm

A subset of ALS-CP samples showed increased C-reactive protein (CRP) compared to controls (Fig. S5). CRP is an acute inflammatory protein synthesized primarily in liver hepatocytes but also by smooth muscle cells, macrophages, endothelial cells, lymphocytes, and adipocytes [69]. CRP plays an active role in the inflammatory process, activating C1q in the complement pathway and depositing in tissues at sites of tissue damage. We examined the localization of CRP in ALS-CP compared to neurological (AD) and non-neurological disease controls. No CRP immunostaining was found in control CP or AD CP. However, ALS-CP had notable increases in CRP immunostaining in 8 of 11 cases (Fig. 11). Intense CRP signal was detected on the inner endothelial walls in most CRP positive ALS cases (red arrowheads), as well as within the blood vessel lumen (possibly on the surface of platelets). We observed some CRP positive signal in the stroma of 3 out of the 8 CRP positive cases examined (yellow arrowhead). In addition, choroid epithelial cells also contained CRP immunoreactivity in some ALS cases (blue arrows).

## Discussion

Although prior studies have explored the BBB/BSCB in ALS, to date none have investigated the BCSFB in human ALS. In the present study, we investigated BCSFB integrity in postmortem CP tissue from patients with ALS and non-neurological controls. We find alterations and/or reductions in expression levels of multiple tight junction and adhesion markers in ALS-CP compared to controls, including Claudin 1 and 3, Occludin and E-cadherin. Interestingly, Claudin 5 which has previously been shown to be down-regulated in medulla and cervical but not lumbar ALS patient spinal cord [25] was not significantly altered in ALS-CP at the mRNA or protein levels. In addition, and similarly to previous reports on ALS BBB/BSCB, we observed down-regulation and discontinuous CD31 expression in endothelial cells in ALS-CP, indicating possible endothelial barrier breakage at the BCSFB (Fig. 7 and [25]). We also report reductions in pericyte numbers around blood vessels in ALS-CP as determined by expression of CD13/ANPEP and PDGFR-β, a phenotype akin to reported pericyte losses in the BSCB in ALS (Figs. 8, S3 and [76]). A dysfunction/damage of the structural elements of the BCSFB (the CP epithelium, the endothelial cells and surrounding pericytes) that typically form a tightly integrated unit regulating access and flow to and from the CNS may contribute to a toxic CNS environment.

Indeed, pericyte losses have been correlated with increased BSCB damage measured by extravascular hemoglobin deposition in human ALS, [76], but also increased BBB permeability to water and large and small molecular weight tracers in mice [4, 18]. In a mouse model of Alzheimer’s disease, pericyte loss increased immunoglobulin G (IgG) extravascular deposition, decreased microvasculature density/capillary length, and increased Aβ deposition and pathology [62]. In the SOD1 ALS mouse model, human pericyte injection extended survival of male mice and activated the host antioxidant response [15]. Pericyte loss is thought to affect brain vessel permeability by modulating endothelial transcytosis as well as deregulating molecular pathways in endothelial cells [3]. Very little is known about the role of pericytes in the CP, although they have been shown to support endothelial cell formation during development [53]. Interestingly, one of the genes deregulated in pericyte-deficient endothelial cells is *VEGFA* [18, 75]. VEGF-A is upregulated in pericyte-deficient states, potentially inducing a disruption of inter-endothelial junctions and increased extracellular matrix (ECM) degradation in endothelial cells, thereby opening the junctional barrier [22, 50].

Interestingly, and consistent with the state of pericyte loss we report, VEGF-A protein is significantly upregulated in ALS-CP samples compared to controls (Fig. 10a). Macrophages have been shown to release large amounts of VEGF-A and affect vascular permeability [39] and MERTK signaling similarly regulates endothelial function and angiogenesis [24, 33]. Both MERTK levels and macrophages along with VEGF-A protein are increased in ALS-CP tissues compared to controls. VEGF-A was previously found to be significantly elevated in serum and CSF of ALS patient [31], but clinical trials boosting VEGF-A levels in ALS patients have failed. Given the effect of VEGF-A on increasing vascular permeability at the various blood-brain and BCSFBs, one can speculate that boosting VEGF-A levels has detrimental effects on BCSFB integrity, thus exacerbating disease pathogenesis.

Increased cytokine levels and decreased tight junction expression at the BBB and BCSF have previously been correlated [5, 12, 42]. In fact, prior studies have shown that endothelial cells exposed to inflammatory cytokines exhibit reduced CD31 expression in intercellular junctions, which enhances T-cell activation [46]. In ALS-CP, we find widespread CD31 loss and a subset of ALS-CP tissues with a high inflammatory signature, as measured by higher levels of IL-6, IL-8, Thrombomodulin and P-Selectin (Fig. 10). These cytokines and chemokines were measured in whole CP tissue lysates that included multiple cell types making the sensitivity of the immunoassays really low, potentially diluting local increases in these cytokines in particular cellular subtypes. It is noteworthy that neither the RNA-seq analysis, nor the immunoassay protein expression data or the immunohistochemical analysis showed remarkable differences between SALS and C9-ALS, suggesting that BCSFB defects are a general mechanism of ALS pathogenesis. We detected few to no dipeptide repeat element proteins (DPRs) (measured by poly GA and poly GP staining) in C9-ALS-CP, and few phospho-TDP43 inclusions, only in the choroidal stroma of 2/12 ALS cases (Fig. S4C and data not shown).

Interestingly, and in contrast to AD models where BCSFB disruptions induced by Aβ oligomers were mediated by MMP3 [11], we observed significant downregulation of all MMP proteins examined (MMP1, 3 and 9) in ALS-CP. MMP9 has previously been shown to be both upregulated [23] and downregulated [52] in CSF of ALS patients, and two studies found it to be increased in ALS serum [7, 19]. MMP1 was unaltered in ALS CSF [52]. It is notable that we did not detect RNA transcripts for either of these MMPs in the RNA-seq in ALS or control tissues, suggesting that the transcriptional source of these proteases is not the CP itself. Nevertheless, the low level of these MMPs in CP may result from impaired balance between the proteases and their inhibitors (TIMPs) and/or their increased degradation/ clearance. Further studies are needed to investigate this observation.

Disruptions in the BCSFB in an ALS mouse model were accompanied by a lack of activation of IFN-γ signaling and a lack of leukocyte trafficking into the CSF and spinal cord [43]. We were unable to detect IFN- γ mRNA in ALS or control tissues and IFN-γ protein was very low/below detection limit in multiple ALS and control samples, although a subset of ALS cases had lower IFN-γ than controls (not reaching statistical significance). Additional inflammatory markers downregulated in the mouse at late disease stages included ICAM-1, CCL2 and Fractalkine. MCP-1/CCL2 and Fractalkine showed a similar trend in ALS-CP tissues, but failed to reach statistical significance. Other notable differences with the SOD1 mouse model include a near absence of T-cells in the CP of either ALS or control groups, although we did observe an accumulation of MERTK positive and Iba1 positive macrophages in ALS-CP. Such differences could be attributed to inherent differences in signaling at the CP level between mice and human, or due to the overexpression of mutant human SOD1 in the transgenic mouse model when compared to sporadic human ALS. In fact, in one ALS patient harboring an SOD1 mutation (N139K), both IFN-γ and ICAM-1 were down-regulated, but Fractalkine was increased (Fig. S5). Nevertheless, the lack of an immune activation response supporting leukocyte trafficking in the CP of the SOD1G93A ALS mouse model [43] is consistent with our human ALS-CP data where we observed significant downregulation of the M-CSF cytokine and the VCAM-1 leukocyte transendothelial migration marker.

An interesting finding in ALS-CP is the induction of platelet activation markers in ALS-CP, shown at the gene expression level from the RNA-seq data and vWF, Fibrinogen and P-selectin immunostaining. Taken together, these indicate a severe underlying vascular injury in CP blood vessels, accompanied by activation of clotting factors. Deposition of vWF and Fibrinogen along inner endothelial walls in ALS but not control CP, along with vWF expression on platelets surface, support this hypothesis (Figs. 4 and 5). No circulating endothelial cells in peripheral blood have been previously detected in moderate or late stage ALS (as defined by ALSFRS measures) [28], suggesting that endothelial damage in ALS is accompanied by repair, presumably through clotting rather than detachment of dysfunctional endothelial cells. It is noteworthy that vWF mRNA was significantly downregulated in ALS-CP, while vWF protein was increased in those tissues suggesting that the source of this factor is not the CP tissue itself, but rather packaged storage α-granules in platelets.

CRP has previously been detected in serum and CSF of ALS patients and its elevation in plasma shown to correlate with faster disease progression [47, 61]. In line with these findings, we have observed higher CRP levels in ALS cases with a higher immune signature as well as higher SAA levels (Fig. S5). Interestingly, at the histological level, we also observed increased CRP levels in the choroid stroma as well as in some CP cells (Fig. 11, blue arrows). This may reflect CRP protein in transit from the plasma compartment towards the CSF through the CP epithelial cells, but it is also possible that CRP itself is expressed by choroidal cells. We also observed a novel accumulation of CRP on the CP endothelial walls of ALS but not control or AD tissues, a phenotype highly reminiscent of atherosclerotic plaques (Fig. 11, red arrowheads). CRP is known to localize directly to atherosclerotic plaques where it induces the expression of genes directly involved in monocytes adhesion, recruitment of intracellular molecules such as E-selectin and activating the complement system [69]. Further study is needed to investigate the nature of the CRP aggregates in ALS-CP.

A cohort of ALS CP samples exhibited an “inflammatory signature” in the protein immunoassay panels (Fig. S5) characterized by elevated levels of multiple cytokines and immunokines, suggesting the existence of a subtype of ALS patients with increased inflammation. This phenotype is highly reminiscent of recent findings in postmortem ALS cortex that described distinct molecular subtypes of ALS, with 19% of assayed cortex samples showing signatures of activated glia and immune cells [71].

Taken together, we hereby describe significant alterations in the BCSFB in ALS, with a disruption of the junctions in the CP epithelial cells, and activation of platelets and immune infiltration into the CP, as well as major vascular disruptions and degradation associated with the disease. Further studies are required to examine markers of CP trans-epithelial transport as well as pathways involved in the metabolic function of these highly energy-consuming cells. Nevertheless, our findings pave the way towards a deeper understanding of the BCSFB involvement and disruption in ALS, and given the CP’s role as one of the drug delivery routes into the CNS could prove highly useful for therapy development and delivery.

## Methods

### Tissue samples

ALS and non-neurologic disease control post-mortem choroid plexus tissue samples were obtained from the Barrow Neurological Institute ALS Tissue Bank, the Target ALS Human Postmortem Tissue Core and the NIH Neurobiobank. All tissues samples were collected after informed consent from the subjects or by the subjects’ next of kin, complying with all relevant ethical regulations. The postmortem tissue bank protocol and consent process were approved by the Dignity Health Institutional Review Board, and both the Target ALS and NIH Neurobiobank use IRB approved protocols and consents to collect and distribute tissue samples for research purposes. Clinical diagnoses were made by board certified neuropathologists according to consensus criteria for ALS. Subject demographics are listed in Suppl. Table 3.

### Bioinformatic methods

Demultiplexing and fastq generation were performed using CASAVA v1.8.4 (Illumina), followed by trimming of 7 nt from both R1 and R2 using cutadapt v1.10, as recommended in the SMARTer manual. Reads were then aligned to the GRCh37 human genome using STAR v2.5.2b, and raw counts were calculated with featureCounts (subread v1.5.1) using the ensembl75 annotation. Analysis and differential expression testing were performed in R using DESeq2 v1.14.1, with batch, sex, and age (< 65 or > 65 years) accounted for in the design. Differentially expressed genes were selected based on the following criteria: adjusted *p*-values (*p* < 0.05), a fold change greater than 2, and a baseMean value greater than 20. Heatmaps were generated using the pheatmap v1.0.8 R package, using Z-transformed values on the r-log transformed expression from DESeq2, after batch correction in limma for flowcell run. For the relaxed parameter analysis, we used: adjusted *p*-values (*p* < 0.1), a fold change greater than 1.5, and a base Mean value greater than 20.

Gene ontology analysis for biological processes and pathways enriched in the dataset was performed using ToppGene Suite using FDR correction and a cutoff of 0.05 [14]. *P*-values were generated using the “Hypergeometric probability mass function”. Biological processes analysis was also performed using DAVID Bioinformatics Resources 6.8 [34]. Gene annotation ontology for biological processes and molecular functions was performed using PANTHER (Protein ANalysis THrough Evolutionary Relationships) Classification System version 14 [51].

### Choroid plexus protein lysate preparation

Fresh frozen tissue was obtained from the Target ALS Postmortem Tissue Core and NIH NeuroBioBank. Frozen tissue was placed into ice-cold lysis buffer (20 mM Tris, pH 7.5, 150 mM NaCl, 1 mM EDTA, 1 mM EGTA, 1% Triton X-100, with 100 uL Phosphatase Inhibitor Cocktail Set II (Sigma) and 100 uL Protease Inhibitor Cocktail (Sigma) added per 10 mL lysis buffer), at an approximate ratio of 67 uL lysis buffer per 10 mg tissue. Lysates were prepared in three batches as tissue was acquired. Tissue was homogenized mechanically with OmniTip probes for hard tissue (Omni International), for 10 s each at low, medium, and high speed. Homogenates were transferred to 1.5 mL microcentrifuge tubes and sonicated in an ice-cold water bath three times for 30 s at 40% amplitude. Samples were rotated at 4 °C for 30 min, and centrifuged at 4 °C for 10 min at 20,000 x g. Supernatant was collected and stored at − 80 °C. Lysate total protein concentration was determined by Bradford assay (Bio-Rad). These lysates were used for western blot analysis using the Invitrogen NuPAGE Bis-Tris protein gels, transferred onto PVDF membranes and probed with primary and secondary antibodies (1:15,000 dilution; LiCor). Signals were imaged by using the Odyssey CLx imager (LiCor).

### Meso scale discovery immunoassay analysis

Twenty-one markers were measured using the following Mesoscale Discovery kits: Human Vascular Injury I Kit, V-PLEX Vascular Injury Panel 2 Human Kit, V-PLEX Human Proinflammatory Panel II + IFN-γ, Human MMP 3-Plex Ultra-Sensitive Kit, and a custom U-PLEX panel for M-CSF, IL-4, MCP-1, Fractalkine, and VEGF-A. Manufacturer instructions were followed for each kit. All samples were bound to plates overnight at 4 °C with vigorous agitation on a MicroMix 5 plate shaker (DPC). Lysates were diluted in the recommended diluent provided with each kit. Of the 21 markers, IL-4 had 65% of the samples below detection limit and ICAM-3 signal was very low and close to background, so neither analyte was included in further analysis.

### RNA extraction and Q-PCR

Total RNA were prepared from frozen tissue from control and ALS cases. Samples were homogenized in Trizol (Invitrogen), and RNA was extracted using the Ambion PureLink™ RNA Mini Kit. RNA quality was determined by RIN (RNA integrity number) using a Tapestation (see Table S4 for RIN values for samples used for RNA-seq). cDNA was synthesized using Superscript VILO (Invitrogen) and real-time RT-PCR was performed using the FastStart Universal SyberGreen master mix (Roche).

### RNA-seq library preparation

Isolated RNA concentrations were measured with Quant-iT Ribogreen RNA Assay (Thermo Fisher, Cat. No.: R11490). For each RNA sample, 100–200 ng total RNA was ribodepleted with Illumina’s Ribo-Zero Gold rRNA Removal Kit (Illumina, Cat. No. MRZG12324) and purified with NucleoSpin RNA XS columns (Machery Nagel, Cat. No. 740902.10) according to Takara Bio’s “Protocol for Removal of rRNA from Small Amounts of Total RNA.” Double-stranded cDNA was synthesized from the ribodepleted RNA using the SMARTer Universal Low Input RNA Kit for Sequencing (Takara Bio, Cat. No. 634940) with a 16-cycle PCR. The concentration of cDNA was measured with Qubit dsDNA HS Reagent (ThermoFisher Scientific, Cat. No. Q32854). Double-stranded cDNA (3.6 - 14 ng) was fragmented with the Covaris E220 (Peak Incident Power = 140 W, Duty Factory = 10%, Cycles/Burst = 200, Treatment Time = 80 s). The fragmented cDNA was then prepared into libraries using KAPA Hyper Prep Kit (KAPA Biosystems, Cat. No. KK8504). This library preparation included a combination end repair & A-tailing reaction, followed by a 4 °C overnight ligation of uniquely barcoded adapters to each sample at a 200:1 adapter to insert molar ratio and a 7-cycle enrichment PCR. The size of each final library was determined by TapeStation (Agilent High Sensitivity D1000 ScreenTape & Sample Buffer, Cat. No. 5067–5584 & Cat. No. 5067–5603), and the concentration was measured with KAPA SYBR FAST Universal qPCR Kit (KAPA Biosystems, Cat. No. KK4824). Libraries were then combined into equimolar pools which were also measured for size and concentration. The pools were clustered onto a paired-end flowcell (Illumina, Cat. No. PE-401-3001) with a 20% v/v PhiX v3 spike-in (Illumina, Cat. No. FC-110-3001) and sequenced on Illumina’s HiSeq 2500 with TruSeq SBS Kit v3-HS chemistry (Illumina, Cat. No. FC-401-3002) to 30 million read pairs per library. The first and second reads were each 83 bases.

### IHC and immunofluorescence

Paraffin-embedded post-mortem tissue sections from choroid plexus were used for this study. All sections were deparaffinized, rehydrated and antigen retrieval performed using Target Antigen Retrieval Solution, pH 9.0 (DAKO) or a citrate buffer (pH 6) for 20 min in a steamer. After cooling to room temperature, non-specific binding sites were blocked using Super Block (Scytek), supplemented with Avidin (Vector Labs). Primary antibodies used for immunohistochemistry were incubated overnight in Super Block with Biotin (antibodies listed in Suppl. Table 5). Slides were then washed and incubated for 1 h in the appropriate biotinylated IgG secondary antibodies (1:200; Vector Labs) in Super Block. Slides were washed in PBS and immunostaining visualized using the Vectastain Elite ABC reagent (Vector Labs) and Vector Immpact NovaRED peroxidase substrate kit (Vector Labs). Slides were counterstained with hematoxylin (Sigma Aldrich).

For immunofluorescence, paraffin sections were deparaffinized, rehydrates and antigen retrieval was performed in appropriate buffers. Sections were blocked in Super Block (Scytek) and primary antibodies were incubated overnight in Antibody Diluent, background reducing (Dako). Slides were then washed in PBS and incubated in Alexafluor-conjugated secondary antibodies, prepared in the same antibody diluent for 1 h at room temperature. Following washes, autofluorescence was decreased using the autofluorescence eliminator (Millipore) for 5 min, and slides were rinsed in 70% ethanol before mounting using Vectashield antifade mounting media with DAPI (Vector labs). Signal intensity was quantified using Imaris and signal intensity was normalized to tissue section volume.

### Statistical analysis

Choroid lysates for the MSD analysis were prepared in three batches, with variable ratios of ALS cases to controls in each batch. To adjust for batch effect, individual markers were compared between ALS and controls, adjusting for batch effects using a likelihood ratio test of deviance. A parent model was fit using logistic regression to predict case/control status from plate number. An extended model was then fit to predict the disease case/control status from the plate number plus the marker of interest. The deviance between the nested models were compared using a likelihood ratio test. *P*-values indicate the significance of a marker’s contributes to the prediction of case/control status after adjusting for plate number. To generate the MSD heat maps, the ComBat R script was used to adjust for batch effect for all markers collectively [38]. Then Z-values across all samples were computed as (value-average/standard deviation).

## Supplementary information

**Additional file 1: Figure S1.** ZO-1 immunofluorescence was performed on control (*n* = 4) and ALS CP (*n* = 5) and staining was imaged under 20x using confocal microscopy. DAPI denotes nuclei and DIC (differential interference contrast) images were taken to show tissue morphology. Scale bar: 50um. Red arrowheads point to areas of loss of ZO-1 staining in between CP epithelial cells. **Figure S2. A)** Immunohistochemistry for Claudin 5 was performed on control (*n* = 3) and ALS CP (*n* = 5). Pictures were taken at 40X magnification. Scale bar: 20 μm. Black Arrows point to staining in endothelial cells and red arrowheads point to staining within the lumen of blood vessels. **B)** Western blot analysis was performed on 9 controls and 18 SALS CP lysates and membranes were probed for Claudin 3 and 5, as well as Occludin. GAPDH was used as a loading control. **Figure S3. A)** Quantification of immunofluorescence signal intensity for CD13 and PDGFRbeta on *n* = 3 control and *n* = 5 ALS CP samples was performed using Imaris. Image stacks were taken at 63x magnification and ten different Z-stack fields per sample were captured and quantified. Tissues boundaries were delineated and signal intensity was normalized to section volume. The asterisk denotes significance with a *p*-value of 0.0095. Values for PDGFRbeta were not found to be significant. **B)** CD13/ANPEP immunohistochemistry in ALS (*n* = 5) and control (*n* = 4) CP. Black arrows point to CD13 stain around blood vessels on controls and red arrowheads denote vascular areas of CD13 loss in ALS. **Figure S4 A)** CD3 staining in ALS-CP tissues. Scale bar: 20 μm. **B)** MERTK immunohistochemistry in ALS and Control CP. Pictures were taken at low magnification to show extent of MERTK expression. Scale bar 100 μm **C)** p-TDP43 (S409/410) staining in CP from ALS and Control postmortem tissues. Only two ALS cases showed any inclusions, and these were located in the choroid stroma. Scale bar: 20 μm. **Figure S5** Heatmap showing Z-scores across all samples for all gene targets that had reliable MSD signal. Samples are presented in their respective groups (control vs ALS), ranked by cumulative sum of all analytes measured. **Figure S6** All cytokines and chemokines analyzed by MSD immunoassays in *n* = 22 controls and *n* = 44 ALS-CP lysates. None of the shown analytes showed significant differences between the two groups through a logistic regression analysis that accounts for patient age (under 65 vs above 65), as well as the plate on which the sample was run to avoid batch effect bias. Both IFN-γ and TNF-α had low signal to background ratio. **Supplemental Table 1.** ToppGene Analysis showing the top 30 enriched Biological Processes. **Supplemental Table 2.** MSD immunoassay analysis of CP lysates from ALS and non-neurological disease controls. **Supplemental Table 3.** Demographics for post-mortem tissue samples. **Supplemental Table 4.** CP samples used for RNA-seq. **Supplemental Table 5.** List of antibodies used in analysis of CP samples.

## Data Availability

The datasets used and/or analysed during the current study are available from the corresponding author on reasonable request.

## Abbreviations

CNS: Central nervous system
CSF: Cerebrospinal fluid
CP: Choroid plexus
BCSFB: Blood-CSF barrier
BBB: Blood-brain barrier
BSCB: Blood-spinal cord barrier
ICAM: Intercellular adhesion molecule
V-CAM: Vascular cell adhesion molecule
AD: Alzheimer’s disease
ALS: Amyotrophic Lateral Sclerosis
