# Supplementary material for: Global alterations to the choroid plexus blood-CSF barrier in amyotrophic lateral sclerosis
Source: Acta Neuropathol Commun. 2020 Jun 26;8:92. doi: 10.1186/s40478-020-00968-9 (PMC7318439; doi:10.1186/s40478-020-00968-9)
Supplement: Supplementary file 1 — Additional file 1: Figure S1. ZO-1 immunofluorescence was performed on control (n = 4) and ALS CP (n = 5) and staining was imaged under 20x using confocal microscopy. DAPI denotes nuclei and DIC (differential interference contrast) images were taken to show tissue morphology. Scale bar: 50um. Red arrowheads point to areas of loss of ZO-1 staining in between CP epithelial cells. Figure S2. A) Immunohistochemistry for Claudin 5 was performed on control (n = 3) and ALS CP (n = 5). Pictures were taken at 40X magnification. Scale bar: 20 μm. Black Arrows point to staining in endothelial cells and red arrowheads point to staining within the lumen of blood vessels. B) Western blot analysis was performed on 9 controls and 18 SALS CP lysates and membranes were probed for Claudin 3 and 5, as well as Occludin. GAPDH was used as a loading control. Figure S3. A) Quantification of immunofluorescence signal intensity for CD13 and PDGFRbeta on n = 3 control and n = 5 ALS CP samples was performed using Imaris. Image stacks were taken at 63x magnification and ten different Z-stack fields per sample were captured and quantified. Tissues boundaries were delineated and signal intensity was normalized to section volume. The asterisk denotes significance with a p-value of 0.0095. Values for PDGFRbeta were not found to be significant. B) CD13/ANPEP immunohistochemistry in ALS (n = 5) and control (n = 4) CP. Black arrows point to CD13 stain around blood vessels on controls and red arrowheads denote vascular areas of CD13 loss in ALS. Figure S4 A) CD3 staining in ALS-CP tissues. Scale bar: 20 μm. B) MERTK immunohistochemistry in ALS and Control CP. Pictures were taken at low magnification to show extent of MERTK expression. Scale bar 100 μm C) p-TDP43 (S409/410) staining in CP from ALS and Control postmortem tissues. Only two ALS cases showed any inclusions, and these were located in the choroid stroma. Scale bar: 20 μm. Figure S5 Heatmap showing Z-scores across all samples for all gene targ [file 40478_2020_968_MOESM1_ESM.pdf]

Figure S1

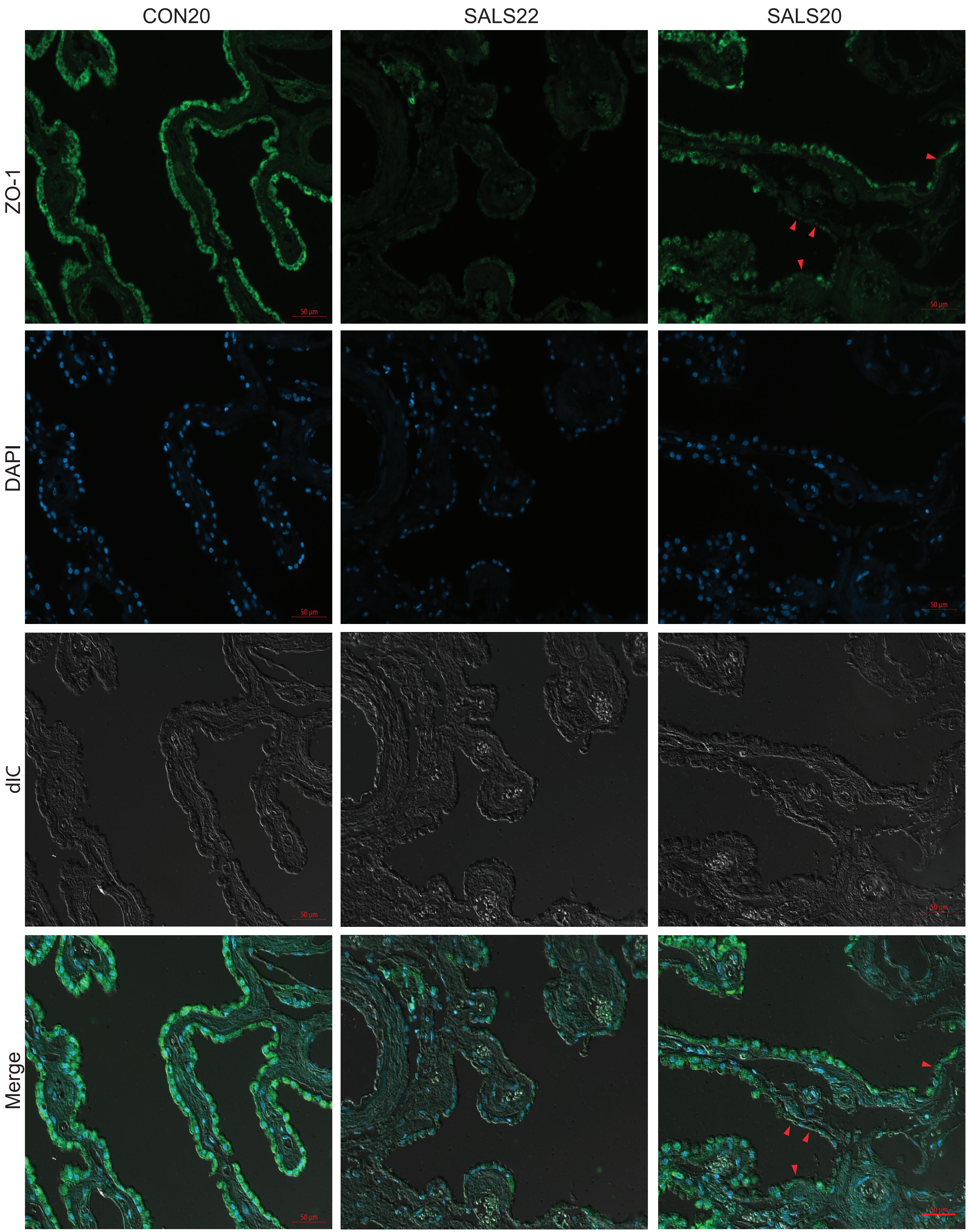

Figure S2

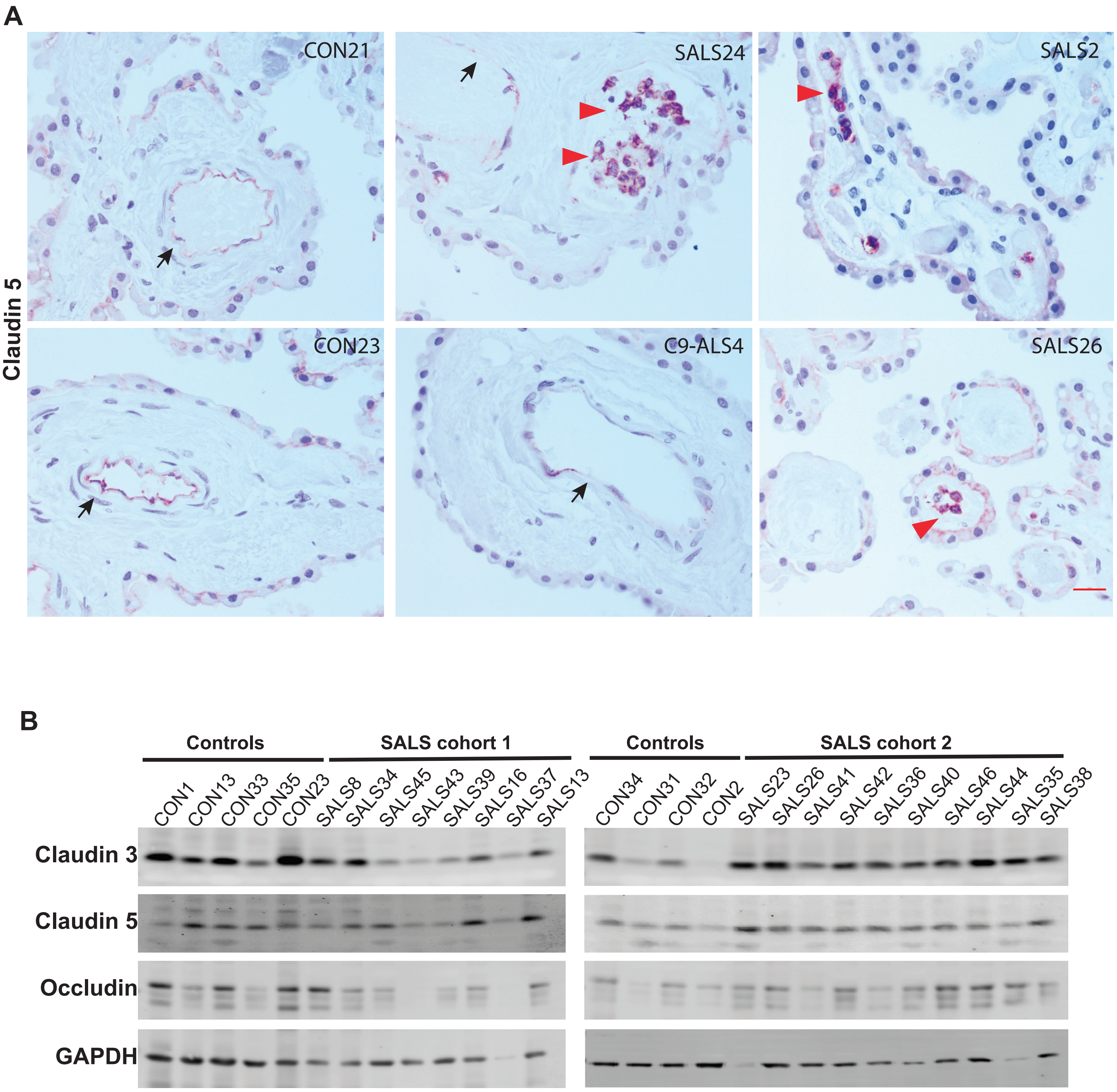
A
Claudin 5
CON21
SALS24
SALS2
CON23
C9-ALS4
SALS26
B
Controls
SALS cohort 1
Controls
SALS cohort 2
CON1
CON13
CON33
CON35
CON23
SALS8
SALS34
SALS45
SALS43
SALS39
SALS16
SALS37
SALS13
CON34
CON31
CON32
CON2
SALS23
SALS26
SALS41
SALS42
SALS36
SALS40
SALS46
SALS44
SALS35
SALS38
Claudin 3
Claudin 5
Occludin
GAPDH

Figure S3

A

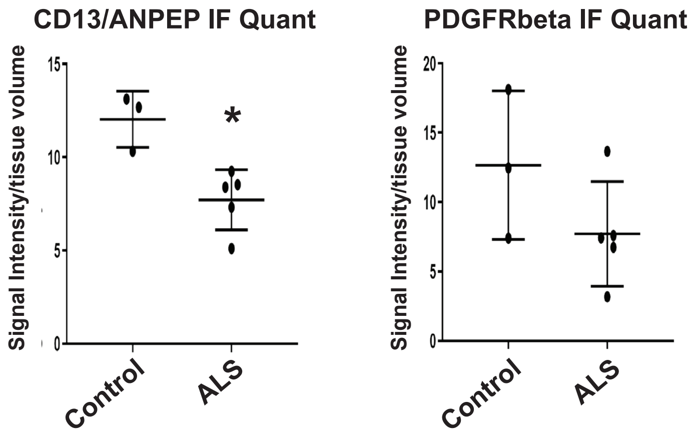
CD13/ANPEP IF Quant
Signal Intensity/tissue volume
15
10
5
0
*
Control
ALS
PDGFRbeta IF Quant
Signal Intensity/tissue volume
20
15
10
5
0
Control
ALS

B

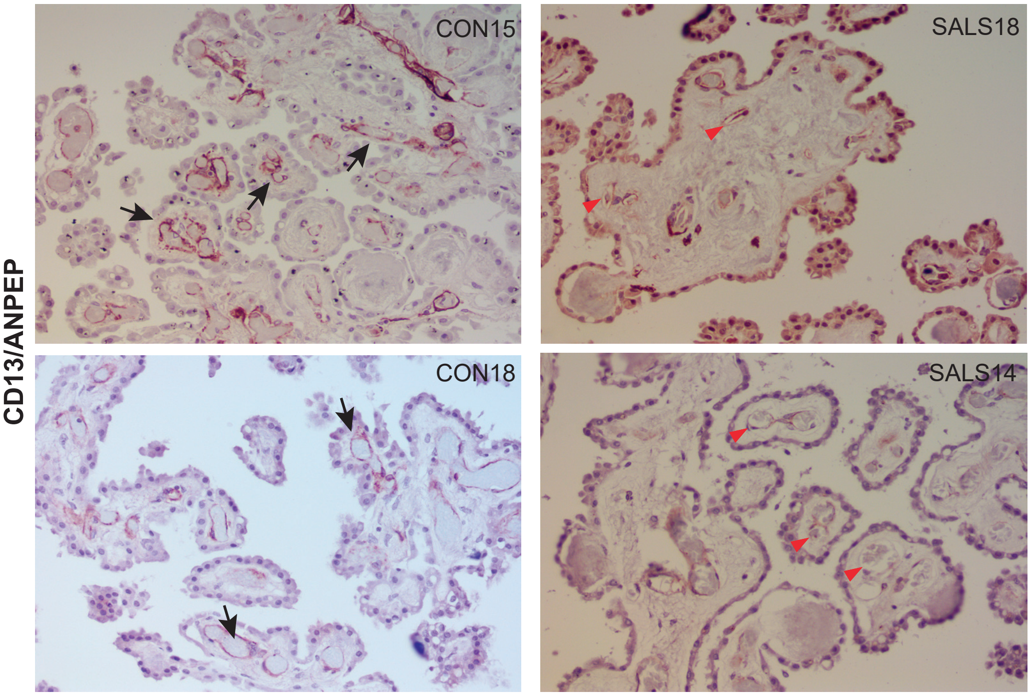
CD13/ANPEP
CON15
SALS18
CON18
SALS14

Figure S4

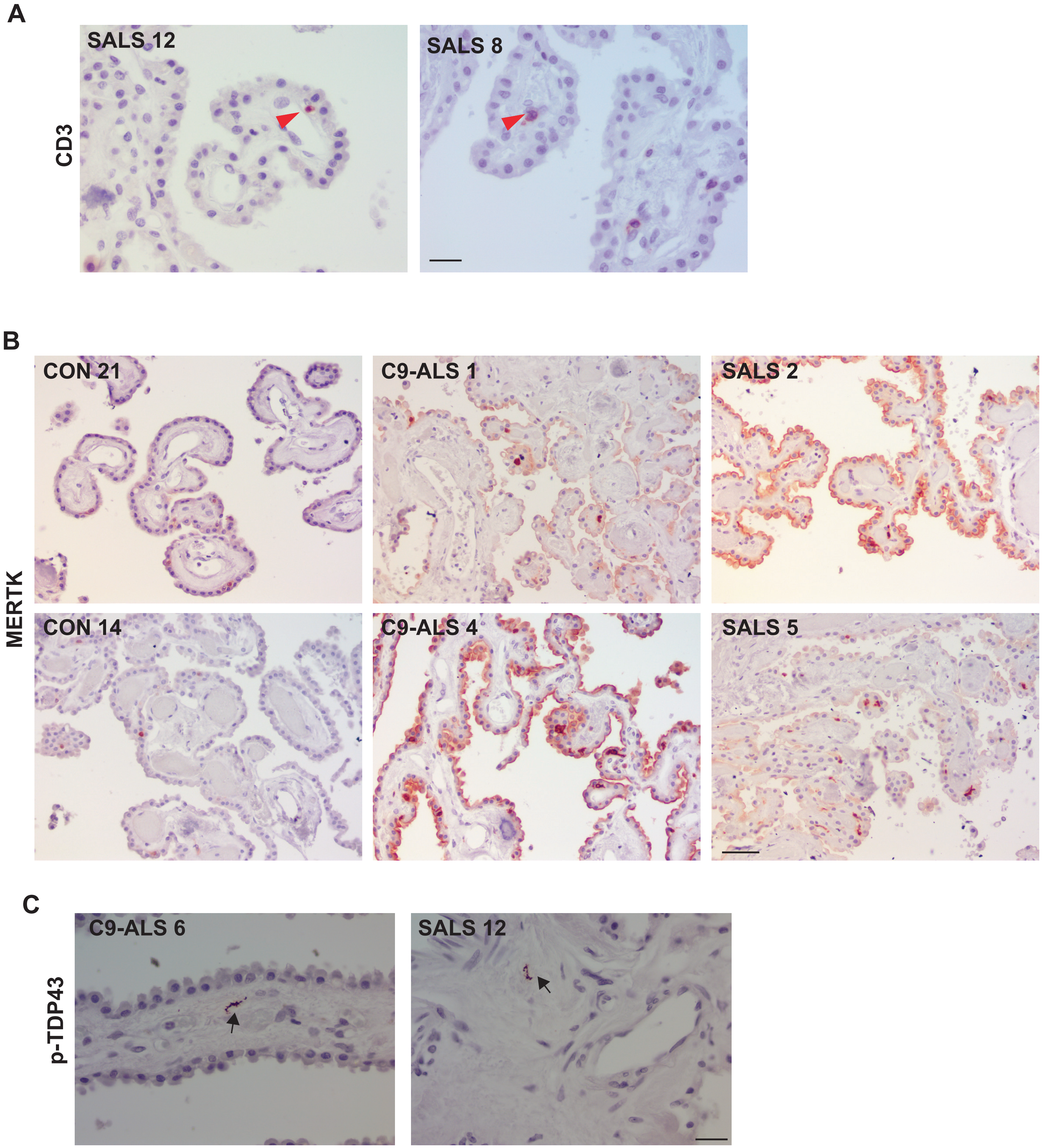

A
CD3
SALS 12
SALS 8
B
MERTK
CON 21
C9-ALS 1
SALS 2
CON 14
C9-ALS 4
SALS 5
C
p-TDP43
C9-ALS 6
SALS 12

Figure S5

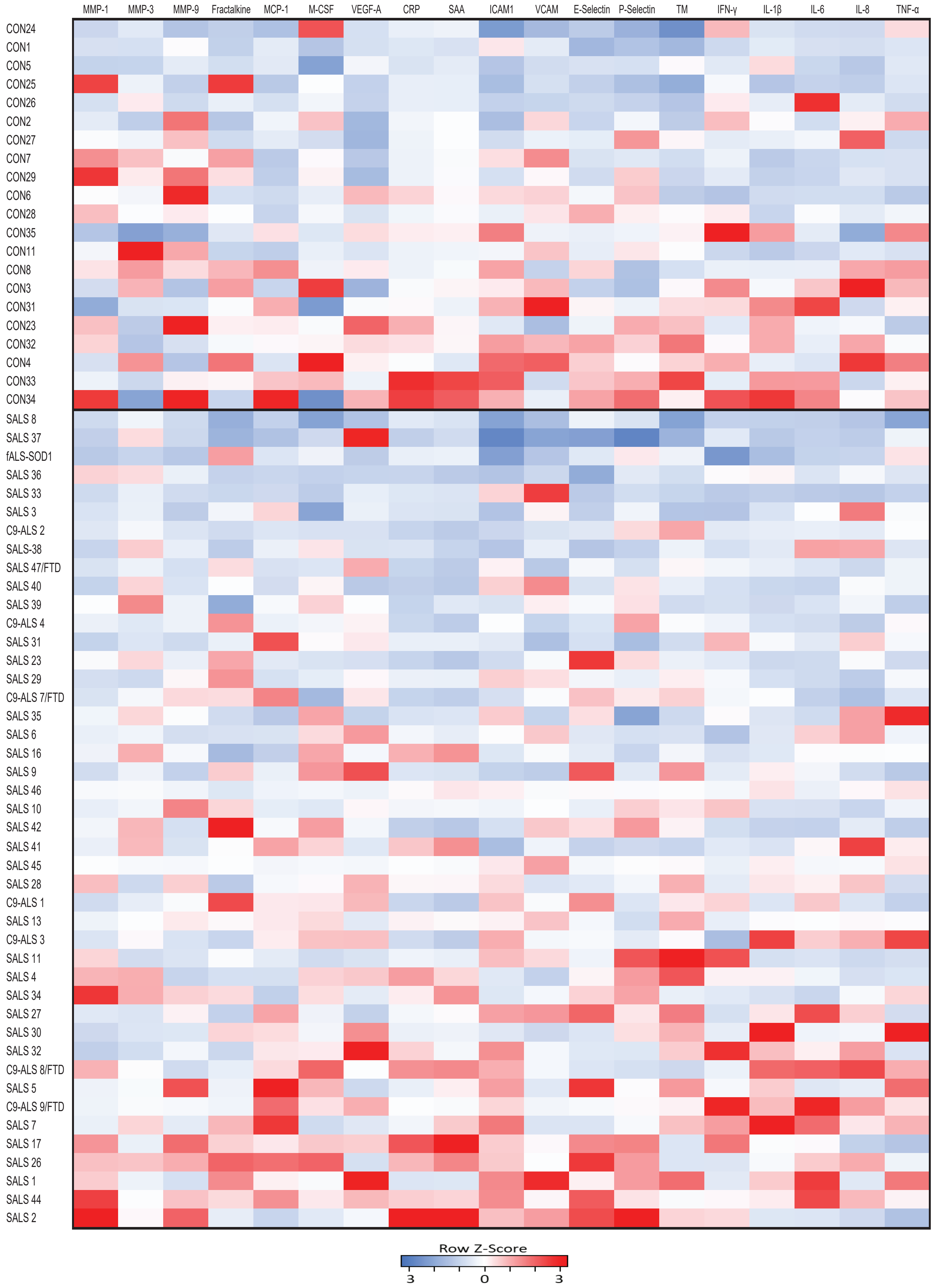
MMP-1
MMP-3
MMP-9
Fractalkine
MCP-1
M-CSF
VEGF-A
CRP
SAA
ICAM1
VCAM
E-Selectin
P-Selectin
TM
IFN-γ
IL-1β
IL-6
IL-8
TNF-α
CON24
CON1
CON5
CON25
CON26
CON2
CON27
CON7
CON29
CON6
CON28
CON35
CON11
CON8
CON3
CON31
CON23
CON32
CON4
CON33
CON34
SALS 8
SALS 37
fALS-SOD1
SALS 36
SALS 33
SALS 3
C9-ALS 2
SALS-38
SALS 47/FTD
SALS 40
SALS 39
C9-ALS 4
SALS 31
SALS 23
SALS 29
C9-ALS 7/FTD
SALS 35
SALS 6
SALS 16
SALS 9
SALS 46
SALS 10
SALS 42
SALS 41
SALS 45
SALS 28
C9-ALS 1
SALS 13
C9-ALS 3
SALS 11
SALS 4
SALS 34
SALS 27
SALS 30
SALS 32
C9-ALS 8/FTD
SALS 5
C9-ALS 9/FTD
SALS 7
SALS 17
SALS 26
SALS 1
SALS 44
SALS 2
Row Z-Score
3
0
3

Figure S6

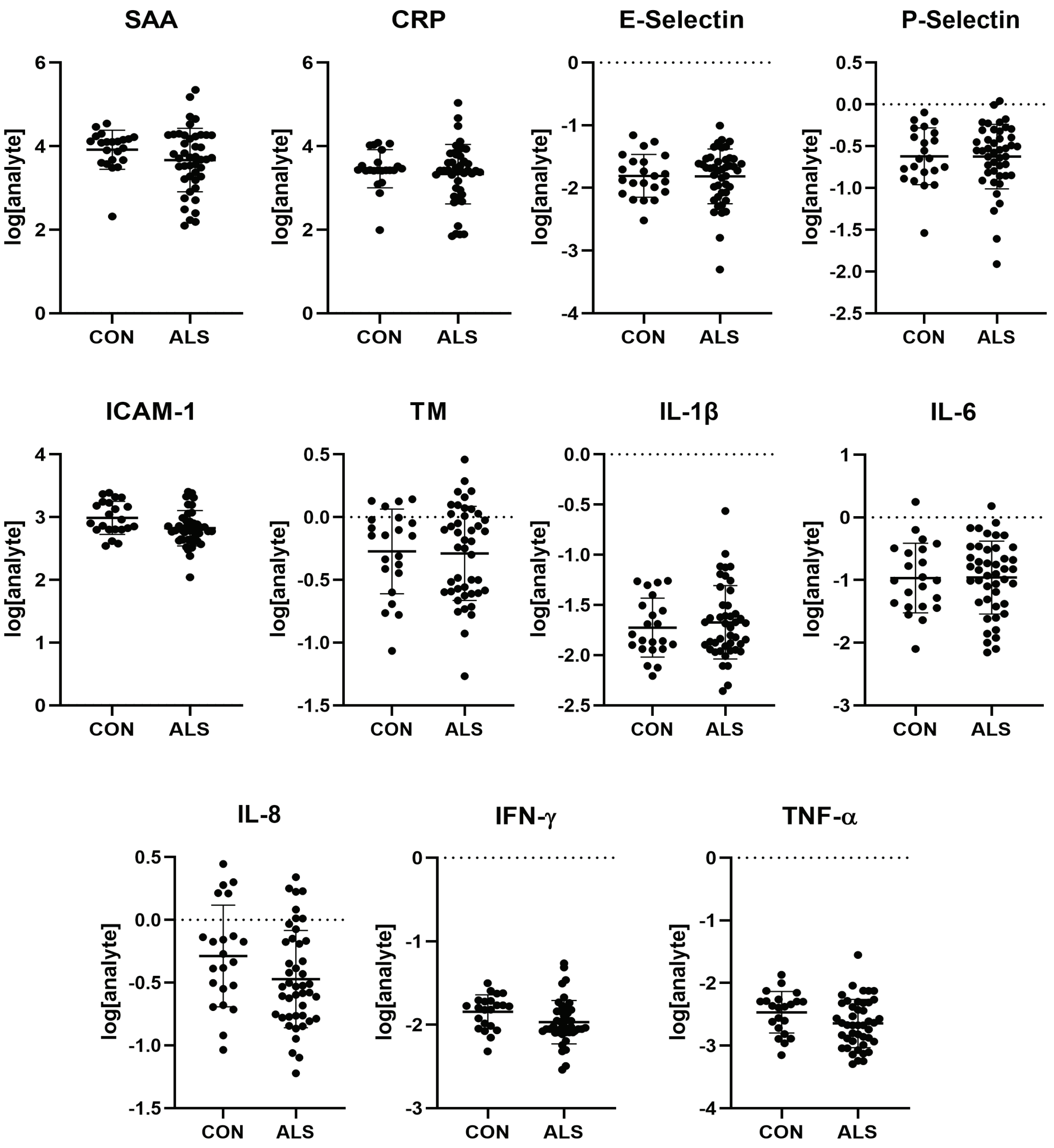

SAA
CRP
E-Selectin
P-Selectin
ICAM-1
TM
IL-1β
IL-6
IL-8
IFN-γ
TNF-α
log[analyte]
CON
ALS
6
4
2
0
-1
-2
-3
-4
0.5
0.0
-0.5
-1.0
-1.5
-2.0
-2.5
3
1

Pathway ID	Name	p-value	q-value Bonferroni	Hit Count in List	Hit Count in Genome	Hit in Query List
GO:0007155	cell adhesion	8.78E-11	9.20E-07	278	1509	ABL1,ACVRL1,ADA,ADAM10,DSCAML1,FZD4,PCDH19,MAD1L1,NLGN2,SRPX,PCDH10,PREX1,JCAD,ANXA2,CNTNAP1,ITGA10,ITGA8,SCARF1,RHOB,ARHGAP6,ARHGDIA,DCHS1,CHRD,ZFHX3,ATP1B1,CTNNAL1,TNFSF14,TNFSF9,AZU1,BGLAP,NRP2,BMP2,BMP6,CDK5R1,ESAM,SART1,CD9,CD27,CD47,CD58,CD151,CDC42,CDH5,CDH6,CDH11,CDH17,CDK6,CEL,AIMP1,SCARF2,CYTH2,CYTH1,KLF4,TAOK2,NRXN2,CLIC1,NTN1,MAP4K4,CNN3,COL1A1,COL3A1,ONECUT2,COL5A1,PRKD2,COL6A1,COL6A2,COL6A3,COL7A1,COL8A1,COL15A1,COL16A1,COMP,MAP3K8,MINK1,CCM2L,PPM1F,SOCS5,CNTNAP2,CYLD,RIPOR2,RC3H1,PIEZO1,SIRPA,DBN1,FARP2,DLG1,DMP1,CDON,DNM2,DGCR2,ITCH,BCL2L11,PTPRU,EFNB1,EFNB2,HMCN1,SORBS3,HSD17B12,B4GALNT2,ENG,ERBB2,FSTL3,ETS1,EMILIN2,LAMC3,ADGRV1,PCDH12,CITED2,PTK2B,BTN2A2,FAT1,MYL9,TESK2,VAV3,GPNMB,FOXF1,FOXC2,SORBS1,NPTN,CGREF1,EGFLAM,CD244,TMEM8B,LRRC32,GATA3,GCNT2,GLI2,GLI3,ADTRP,FREM3,EMILIN1,GSN,CDC42EP1,FAM107A,HAS1,HLADMB,RIC8A,HSPB1,HSPD1,HSPG2,IBSP,ID1,IFNG,IGF2,IHH,IL4,IL4R,IL6,IL6R,IL15,ISLR,ITGA3,ITGA9,LAMA2,LRP1,BCAM,SMAD6,DACT2,MCAM,CD46,MEN1,MELTF,CD99,MKLN1,AFDN,MYO1F,NEXMIF,FXYD5,FGFRL1,UNC13D,CCDC88B,NEO1,NF2,NID1,NODAL,VWA2,NOTCH1,PNP,CNTN4,NPY2R,DDR2,CLDN11,PCDH1,PCDHGC3,PCDH9,PCSK5,PDCD1,SERPINB8,PLD2,PLXNA1,APBB1IP,PML,ROBO4,EPB41L4B,PODXL,MXRA8,PRKAR1A,PRKCE,MAPK7,FBLIM1,ASTL,PTPN2,PTPRC,PTPRF,PTPRS,LYPD5,NECTIN1,NECTIN2,PXN,NDNF,RARA,PRPH2,MMRN2,S100A9,SCN1B,CCL19,XCL1,SDC4,PEAR1,SELE,PLXNA3,ITGA11,STAB2,IFT74,SHC1,NTNG1,ADGRL1,C2CD4A,NLGN1,SIPA1,SLC4A1,LIMS2,RADIL,PARVA,SIGLEC1,SPTA1,SRC,NFASC,PLXND1,SMOC1,PARVG,SSPO,VSIR,STAB1,TINAGL1,STK10,CNTNAP5,ROBO3,CARMIL2,PCDHGA10,PCDHGA5,PCDHGA4,PCDHGA2,NFKBIZ,TFE3,ITGB3BP,TGFB1,TGM2,THBS3,TLN1,PARD3,TNXB,CD2AP,TSC2,COL14A1,VAV1,VEGFA,VWF,P2RY12,WNT5A,AKIP1,PCDH20,CLDN15,DOCK8,RTN4,ZMIZ1,ADAM12,SYMPK
GO:0022610	biological adhesion	9.06E-11	9.50E-07	279	1516	ABL1,ACVRL1,ADA,ADAM10,DSCAML1,FZD4,PCDH19,MAD1L1,NLGN2,SRPX,PCDH10,PREX1,JCAD,ANXA2,CNTNAP1,ITGA10,ITGA8,SCARF1,RHOB,ARHGAP6,ARHGDIA,DCHS1,CHRD,ZFHX3,ATP1B1,CTNNAL1,TNFSF14,TNFSF9,AZU1,BGLAP,NRP2,BMP2,BMP6,CDK5R1,ESAM,SART1,CD9,CD27,CD47,CD58,CD151,CDC42,CDH5,CDH6,CDH11,CDH17,CDK6,CEL,AIMP1,SCARF2,CYTH2,CYTH1,KLF4,TAOK2,NRXN2,CLIC1,NTN1,MAP4K4,CNN3,COL1A1,COL3A1,ONECUT2,COL5A1,PRKD2,COL6A1,COL6A2,COL6A3,COL7A1,COL8A1,COL15A1,COL16A1,COMP,MAP3K8,MINK1,CCM2L,PPM1F,SOCS5,CNTNAP2,CYLD,RIPOR2,RC3H1,PIEZO1,SIRPA,DBN1,FARP2,DLG1,DMP1,CDON,DNM2,DGCR2,ITCH,BCL2L11,PTPRU,EFNB1,EFNB2,HMCN1,SORBS3,HSD17B12,B4GALNT2,ENG,ERBB2,FSTL3,ETS1,EMILIN2,LAMC3,ADGRV1,PCDH12,CITED2,PTK2B,BTN2A2,FAT1,MYL9,TESK2,VAV3,GPNMB,FOXF1,FOXC2,SORBS1,NPTN,CGREF1,EGFLAM,CD244,TMEM8B,LRRC32,GATA3,GCNT2,GLI2,GLI3,ADTRP,FREM3,EMILIN1,GSN,CDC42EP1,FAM107A,HAS1,HLADMB,RIC8A,HSPB1,HSPD1,HSPG2,IBSP,ID1,IFNG,IGF2,IHH,IL4,IL4R,IL6,IL6R,IL15,INHBB,ISLR,ITGA3,ITGA9,LAMA2,LRP1,BCAM,SMAD6,DACT2,MCAM,CD46,MEN1,MELTF,CD99,MKLN1,AFDN,MYO1F,NEXMIF,FXYD5,FGFRL1,UNC13D,CCDC88B,NEO1,NF2,NID1,NODAL,VWA2,NOTCH1,PNP,CNTN4,NPY2R,DDR2,CLDN11,PCDH1,PCDHGC3,PCDH9,PCSK5,PDCD1,SERPINB8,PLD2,PLXNA1,APBB1IP,PML,ROBO4,EPB41L4B,PODXL,MXRA8,PRKAR1A,PRKCE,MAPK7,FBLIM1,ASTL,PTPN2,PTPRC,PTPRF,PTPRS,LYPD5,NECTIN1,NECTIN2,PXN,NDNF,RARA,PRPH2,MMRN2,S100A9,SCN1B,CCL19,XCL1,SDC4,PEAR1,SELE,PLXNA3,ITGA11,STAB2,IFT74,SHC1,NTNG1,ADGRL1,C2CD4A,NLGN1,SIPA1,SLC4A1,LIMS2,RADIL,PARVA,SIGLEC1,SPTA1,SRC,NFASC,PLXND1,SMOC1,PARVG,SSPO,VSIR,STAB1,TINAGL1,STK10,CNTNAP5,ROBO3,CARMIL2,PCDHGA10,PCDHGA5,PCDHGA4,PCDHGA2,NFKBIZ,TFE3,ITGB3BP,TGFB1,TGM2,THBS3,TLN1,PARD3,TNXB,CD2AP,TSC2,COL14A1,VAV1,VEGFA,VWF,P2RY12,WNT5A,AKIP1,PCDH20,CLDN15,DOCK8,RTN4,ZMIZ1,ADAM12,SYMPK
GO:0030029	actin filament-based process	1.05E-10	1.10E-06	171	829	ABL1,FMNL2,ADD1,TTC8,ADRB1,PREX1,FGD2,RHOB,RHOG,ARHGAP6,ARHGDIA,ARRB1,PHACTR1,MYOM1,CDK5R1,CACNA1H,CACNA1G,CACNA1C,CACNA2D1,CAMK2B,CAPZA1,CAPZA2,CASQ1,FMNL3,INA,CD47,ARHGEF2,CDC42,PDLIM7,CFL2,CYTH2,TAOK2,MYOZ1,SUN2,LMOD1,CNN3,MINK1,KANK2,PPM1F,CTSV,NEURL2,AIF1L,SIRPA,DBN1,ARHGEF17,ELMO1,STAP1,FARP2,DES,FCHSD2,VILL,DIAPH2,DLG1,DPYSL3,WASHC3,ACTR2,AKAP9,WASF2,SORBS3,EP300,EPB41L2,F2RL1,PTK2B,FAT1,RAPGEF3,TESK2,AMOTL2,FLNC,SORBS1,ANKRD1,FOXP1,SPIRE2,MYOZ2,GMFB,TMSB15A,GSN,CDC42EP1,LDB3,FAM107A,KANK3,CORO6,PDCD10,PACSIN2,SYNPO,ID1,SHANK3,SCIN,KLHL17,KCNE1,KCNJ2,KRT19,LCP1,ARHGAP12,LRP1,ARPIN,MKLN1,MYOM3,FHOD1,MYO1F,MYH6,MYH11,MYL1,MYL3,MYO1C,MYO1E,MYO6,MYO9B,NF2,FGD5,NOTCH2,NRAP,NTRK3,RFLNA,PDGFB,PDGFRB,WIPF3,PLS1,PRICKLE4,ARHGEF19,EPB41L4B,PACSIN1,SH3KBP1,PRKAR1A,PRKCE,PRKCI,TRPM4,PXN,ARHGAP28,EHD2,S100A9,SCN1B,SCN4B,SDC4,SELE,SHC1,MYO18A,ARHGEF15,SMTN,PARVA,FGD6,SPTA1,SPTAN1,SRC,PARVG,STC1,RHOBTB2,SYNE2,PDLIM2,CARMIL2,OBSL1,THSD7B,TESK1,TGFB1,RHOQ,TLN1,TMOD1,DAAM2,PARD3,TNXB,TPM2,SYNPO2,CD2AP,TRPM2,SH3BP1,MICAL1,WIPF1,FMN2,PDXP,FGD3,FCHSD1,NUAK2
GO:0030036	actin cytoskeleton organization	1.88E-09	1.98E-05	148	717	ABL1,FMNL2,ADD1,TTC8,PREX1,FGD2,RHOB,RHOG,ARHGAP6,ARHGDIA,ARRB1,PHACTR1,MYOM1,CDK5R1,CAMK2B,CAPZA1,CAPZA2,CASQ1,FMNL3,INA,CD47,ARHGEF2,CDC42,PDLIM7,CFL2,CYTH2,TAOK2,MYOZ1,LMOD1,CNN3,MINK1,KANK2,PPM1F,CTSV,NEURL2,AIF1L,SIRPA,DBN1,ARHGEF17,ELMO1,STAP1,FARP2,FCHSD2,VILL,DIAPH2,DLG1,DPYSL3,WASHC3,ACTR2,WASF2,SORBS3,EP300,EPB41L2,F2RL1,PTK2B,FAT1,RAPGEF3,TESK2,AMOTL2,SORBS1,ANKRD1,FOXP1,SPIRE2,MYOZ2,GMFB,TMSB15A,GSN,CDC42EP1,LDB3,FAM107A,KANK3,CORO6,PDCD10,PACSIN2,SYNPO,ID1,SHANK3,SCIN,KLHL17,KRT19,LCP1,ARHGAP12,LRP1,ARPIN,MKLN1,MYOM3,FHOD1,MYO1F,MYH6,MYH11,MYO1C,NF2,FGD5,NOTCH2,NRAP,NTRK3,RFLNA,PDGFB,PDGFRB,WIPF3,PLS1,PRICKLE4,ARHGEF19,EPB41L4B,PACSIN1,SH3KBP1,PRKAR1A,PRKCE,PRKCI,PXN,ARHGAP28,EHD2,S100A9,SDC4,SHC1,MYO18A,ARHGEF15,SMTN,PARVA,FGD6,SPTA1,SPTAN1,SRC,PARVG,RHOBTB2,PDLIM2,CARMIL2,OBSL1,THSD7B,TESK1,TGFB1,RHOQ,TLN1,TMOD1,DAAM2,TNXB,TPM2,SYNPO2,CD2AP,TRPM2,SH3BP1,MICAL1,WIPF1,FMN2,PDXP,FGD3,FCHSD1,NUAK2
GO:0042692	muscle cell differentiation	4.20E-09	4.40E-05	103	455	ABL1,ACADM,ARID1A,ADRB1,TSHZ3,ITGA8,CAMK1,ARRB2,HAMP,MYOM1,TNFSF14,STAC3,HDAC3,BMP2,KLF5,CACNA1H,CACNB4,CASQ1,CCNT2,CD9,CDC42,CDK9,CACNA2D2,CFL2,MYOZ1,LMOD1,HOMER1,COMP,GDF15,HDAC9,RIPOR2,NEURL2,HDAC4,MAML1,MYEF2,DMPK,CDON,HDAC5,RCAN1,MYOF,EFNB2,EIF5A,ENG,PLEKHO1,EP300,SPEG,FOXF1,FLNC,FLT3LG,WFIKKN1,ANKRD1,FOXP1,G6PD,MYOZ2,GATA6,EHD1,PLPP7,LDB3,IFRD1,IGF2,IL4,IL4R,CXCL10,KRT19,SMAD6,FOXO4,MYOM3,MYH6,MYH11,NEO1,NFATC4,NOTCH1,NRAP,PDGFB,PDGFRB,MIR140,PRKAR1A,MAPK11,SEMA4C,RARA,TMEM204,RORA,RXRA,RYR1,EHD2,MAPK12,SGCB,SKI,SMARCD3,SUPT6H,TBX1,TBX2,OBSL1,TGFB1,HEY1,HEY2,TMOD1,HIRA,COL14A1,SMYD3,VEGFA,CYP26B1,ADAM12

GO:0007264	small GTPase mediated signal transduction	5.71E-09	5.98E-05	126	594	ABCA1,LZTR1,ABL1,ADRB1,ARHGAP31,DOCK6,PREX1,ARHGAP21,FGD2,DGKZ,APOE,MADD,RHOB,RHOG,ARHGAP6,ARHGDIA,ARL3,ARRB1,USO1,RAB40C,CTNNAL1,RAB11A,CBL,KRIT1,USP8,ARHGEF1,DGKI,ARHGEF2,CDC42,RAB11B,RASGRP4,CYTH2,CYTH1,CHM,SHC2,NTN1,MAP4K4,COL1A2,COL3A1,RAB3D,KANK2,DEPDC7,RIPOR2,STARD8,ARHGEF17,ELMO1,FARP2,G3BP2,DENND4B,CDON,DNM2,TRIM28,LPAR6,WASF2,TNK2,RASGRP2,SPRY3,ERBB2,DNAJC27,GMIP,F2RL1,F2RL2,RAB6C,RAPGEF3,VAV3,HACD3,RASL10A,GNA13,CYTH4,ARPP19,GDI2,RALBP1,GNA12,GNB1,MAPRE2,GPR17,CDC42EP1,PDCD10,RERG,ARHGAP30,ITGA3,KPNB1,ARHGAP12,MYO9B,RAB4B,FGD5,NOTCH1,NOTCH2,PDGFRB,PLD2,ARHGEF19,MAPK11,RIPOR1,RAB4A,RAP1B,ARHGAP28,TAX1BP3,RASA3,SHC1,SIPA1,ARHGEF15,RAB18,ARHGEF40,RHEBL1,FGD6,ARHGAP15,SRC,RHOBTB2,RAB17,RHOQ,ARAP3,TIMP2,ARHGAP45,TP53,CD2AP,SH3BP1,TSC2,NUP62,VAV1,IFT22,FGD3,DOCK8,RTN4,RASGEF1A,SHOC2,SPATA13
GO:0061061	muscle structure development	1.19E-08	1.25E-04	155	780	ABL1,ACADM,ARID1A,ADRB1,TSHZ3,ITGA8,CAMK1,ARRB2,ZFHX3,HAMP,MYOM1,TNFSF14,STAC3,HDAC3,BMP2,KLF5,CACNA1H,CACNB4,CASQ1,CCNT2,CD9,CDC42,CDK9,CACNA2D2,PDLIM7,CFL2,COPS2,MYOZ1,LMOD1,HOMER1,COL3A1,COL6A3,COL11A1,COMP,GDF15,SOSTDC1,CCM2L,CMTM5,HDAC9,RIPOR2,NEURL2,HDAC4,MAML1,MYEF2,DES,DMPK,CDON,HDAC5,RCAN1,TSC22D3,HEYL,MYOF,EFNB2,EIF5A,ENG,PLEKHO1,EP300,ERBB4,SPEG,EVC,CITED2,FHL1,FOXF1,FOXC1,FOXC2,FLNC,FLT3LG,UQCC2,SIRT6,HDAC7,WFIKKN1,ANKRD1,FOXP1,G6PD,MYOZ2,GATA6,EHD1,VAX1,PLPP7,LDB3,HMGCR,ALX4,ID3,IFRD1,IGF2,IGFBP3,IL4,IL4R,IL6,CXCL10,KRT19,LAMA2,LY6E,SMAD6,MEF2D,FOXO4,MYOM3,BCL9L,MYH6,MYH11,MYL3,MYLK,FGFRL1,NEO1,NFATC4,NOTCH1,NRAP,PDGFB,PDGFRB,PRICKLE4,MIR140,PPP2R3A,RANBP3L,PRKAA1,RBFOX1,PRKAR1A,MAPK11,SEMA4C,RARA,TMEM204,RORA,MEG3,RXRA,RYR1,EHD2,MAPK12,ITGA11,SGCA,SGCB,SGCG,SKI,HIF1AN,SMTN,SMARCD3,SOX11,UNC45A,SUPT6H,PDLIM2,TBX1,TBX2,TCF21,OBSL1,NR2F2,TGFB1,HEY1,HEY2,TMOD1,HIRA,COL14A1,SMYD3,VEGFA,CYP26B1,WNT5A,JPH2,ADAM12
GO:0060348	bone development	1.67E-08	1.75E-04	64	246	ALPL,ANXA2,SCUBE2,DCHS1,BGLAP,BMP2,BMP6,SERPINH1,CBS,DHRS3,CHAD,LRP5L,COL1A1,COL6A1,COL6A2,COL6A3,COL7A1,COMP,OSR2,SLC38A10,WASF2,ENG,EP300,LRRC17,SPNS2,CITED2,FGFR3,SCARA3,FOXC1,CARM1,FOXP1,GLG1,GLI3,HSPG2,IHH,LTBP3,MATN1,MEF2D,VWA2,NOTCH2,RFLNA,RANBP3L,PTPRC,RARA,RARG,LRRK1,RYR1,CTC1,CHSY1,SKI,SRC,STC1,SULF2,PHOSPHO1,NBEAL2,CCDC154,PDGFC,TGFB1,THBS3,THPO,TP53,COL14A1,CYP26B1,VWA1
GO:0072359	circulatory system development	1.98E-08	2.07E-04	238	1323	ABL1,ACADM,ACVRL1,ARID1A,ADAM10,ADD1,FZD4,ADRB1,GRK2,JCAD,TEAD2,ANPEP,ANXA2,PIK3R3,APOB,APOE,LMO4,RHOB,ARRB2,DCHS1,HAMP,DNAH11,MYOM1,ADGRB2,BAX,NRP2,HDAC3,BMP2,KLF5,CACNA1C,CAD,KRIT1,FMNL3,DAW1,CDC42,CDH5,DHRS3,AIMP1,PDLIM7,CHM,KLF4,LRP5L,COL1A1,COL1A2,COL3A1,COL5A1,PRKD2,COL8A1,COL11A1,COL15A1,COMP,ADGRA2,ATF2,COL23A1,FOXP4,CCM2L,CSPG4,PPP1R16B,HDAC9,ISM1,MAML1,CUL7,TMEM65,DLX3,MED12,DNM2,SLC12A6,HDAC5,E2F2,ECE1,HEYL,EFNB2,WASF2,CALCRL,ENG,MTDH,EP300,ERBB2,ERBB4,SPEG,ETS1,WARS2,KLF2,CITED2,PTK2B,RAPGEF3,VAV3,GPNMB,AMOTL2,FOXF1,FOXC1,PRRX2,FOXC2,FLT4,ZDHHC16,SIRT6,HDAC7,GNA13,ANKRD1,FOXP1,SUFU,G6PD,GAA,GATA2,GATA3,GATA6,PPP1R13L,GJA4,GLI2,GLI3,TMED2,GNA11,GRN,ADTRP,EMILIN1,LDB3,PDCD10,RIC8A,HSPB1,HSPG2,ID1,ID3,IFNG,IGF2,HSPB6,IHH,IL6,IL6R,CXCL10,ITGA3,LRP1,LY6E,SMAD6,MBD1,MCAM,MEF2D,MAP3K3,MIR2355,FOXO4,MYOM3,MYH6,MYH11,MYL3,MYLK,MYO1E,RIPPLY3,FGFRL1,NDP,NFATC4,NFE2L2,NODAL,NOTCH1,NOTCH2,NOTCH3,NOTCH4,NPY2R,NPY5R,NRAP,NTRK3,OXCT1,OXTR,PCSK5,PDGFB,PDGFRB,SOX18,CARD10,PGF,PIK3C2A,PDCL3,PLCD1,PML,ROBO4,PRICKLE4,EFEMP2,MIR140,MIR16-2,CLEC14A,PRKAR1A,MAPK7,MAPK11,MIR34C,PTGIS,PXN,NDNF,RARA,TMEM204,DYNC2H1,KIF7,MMRN2,RORA,RXRA,RYR1,STAB2,NAA15,SGCB,IFT74,SGCG,SHC1,HIF1AN,ARHGEF15,SCUBE1,SLIT3,PARVA,SMARCD3,KDM2A,SNAI1,ADAP2,SOX11,SP1,TAB2,PLXND1,STAB1,SMG9,PDLIM2,TBX1,TBX2,TBXA2R,TCF21,OBSL1,HIF3A,NR2F2,TGFB1,TIE1,HEY1,HEY2,TNFAIP2,TP53,TSC2,COL14A1,VEGFA,WNT5A,SPHK2,APOLD1,RTN4,JPH2,ZMIZ1,ADAM12,NPRL3
GO:0051056	regulation of small GTPase mediated signal transduction	2.94E-08	3.09E-04	83	355	ABCA1,LZTR1,ABL1,ARHGAP31,PREX1,ARHGAP21,FGD2,DGKZ,APOE,MADD,RHOB,RHOG,ARHGAP6,ARHGDIA,ARRB1,CBL,ARHGEF1,DGKI,ARHGEF2,CDC42,RASGRP4,CYTH2,CYTH1,MAP4K4,COL3A1,KANK2,DEPDC7,RIPOR2,STARD8,ARHGEF17,FARP2,DENND4B,CDON,DNM2,LPAR6,SPRY3,ERBB2,GMIP,F2RL1,F2RL2,VAV3,GNA13,CYTH4,ARPP19,GDI2,RALBP1,MAPRE2,GPR17,PDCD10,ARHGAP30,ITGA3,ARHGAP12,MYO9B,FGD5,NOTCH1,NOTCH2,PDGFRB,ARHGEF19,RIPOR1,ARHGAP28,RASA3,SHC1,SIPA1,ARHGEF15,ARHGEF40,FGD6,ARHGAP15,SRC,RHOBTB2,RHOQ,ARAP3,TIMP2,ARHGAP45,CD2AP,SH3BP1,TSC2,NUP62,VAV1,FGD3,RTN4,RASGEF1A,SHOC2,SPATA13
GO:0048646	anatomical structure formation involved in morphogenesis	3.51E-08	3.68E-04	243	1365	ABL1,ACVRL1,ARID1A,ADA,ADD1,FZD6,SLC24A4,JCAD,TEAD2,ANPEP,ANXA2,PIK3R3,CNTNAP1,ITGA8,CAMK1,LMO4,AR,RHOB,DCHS1,CHRD,MYOM1,TNFSF14,ADGRB2,NRP2,BMP2,CDK5R1,KLF5,CACNA1H,CASP9,CASQ1,KRIT1,FMNL3,CCNG1,CD9,CDC42,CDH5,AIMP1,CFL2,CHAD,KLF4,MYOZ1,LMOD1,LRP5L,COL1A1,COL5A1,PRKD2,COL6A1,COL7A1,COL8A1,COL11A1,COL15A1,GDF15,OSR2,ADGRA2,ATF2,COL23A1,SH3PXD2A,CSPG4,PPP1R16B,HDAC9,RIPOR2,NEURL2,RC3H1,ISM1,FIG4,DLD,MAFB,DMP1,MED12,CDON,SLC12A6,HDAC5,BCL2L11,E2F2,HEYL,MYOF,EFNB2,WASF2,CALCRL,ENG,PLEKHO1,MTDH,EP300,ERBB2,ETS1,TCIRG1,EXT2,EYA2,KLF2,CITED2,PTK2B,DACT1,RAPGEF3,VAV3,GPNMB,AMOTL2,FOXF1,FOXC1,FOXC2,FLT3LG,FLT4,SIRT6,HDAC7,GNA13,SF3B6,ANKRD1,FOXP1,SUFU,GATA2,GATA3,MYOZ2,GATA6,GLI2,GLI3,EHD1,TMED2,CECR2,GRN,ADTRP,EMILIN1,LDB3,ATOH8,PDCD10,RIC8A,HSPB1,HSPG2,ID1,SHANK3,IGF2,HSPB6,IHH,IL4,IL4R,IL6,CXCL10,ITGA3,KRT19,LFNG,TM4SF1,CD109,MCAM,MAP3K3,MIR2355,MFNG,FOXO4,MMP8,MYOM3,MYH6,MYH11,UNC13D,NEO1,NF2,NFATC4,NFE2L2,NODAL,CNOT2,NOTCH1,NOTCH2,NOTCH3,NOTCH4,NRAP,PAX2,PDGFRB,SOX18,CARD10,ATP8B1,PGF,PIK3C2A,PDCL3,PLCD1,PML,ROBO4,PODXL,IRX3,IRX6,WDR74,PPP2R3A,MIR16-2,CLEC14A,PRKAR1A,MAPK7,FAM20A,MIR34C,PTGIS,SEMA4C,NECTIN1,NECTIN2,PXN,CDC73,NDNF,RARA,RARG,MMRN2,RORA,RXRA,EHD2,SDC4,STAB2,NAA15,SHC1,SKI,PARVA,SMARCD3,KDM2A,SNAI1,SOX11,SP1,NFASC,PLXND1,ST14,STAB1,SUPT6H,NBEAL2,TBX1,TBX2,TBXA2R,TCF21,TCOF1,OBSL1,HIF3A,TGFB1,TGM2,THBS3,TIE1,HEY1,TJP1,HEY2,TMF1,TMOD1,TNFAIP2,TP53,TSC2,HIRA,RPL7L1,CCDC136,VEGFA,WNT5A,WNT9B,TXNRD3,APOLD1,FZD3,RTN4,ADAM12
GO:1901701	cellular response to oxygen-containing compound	4.14E-08	4.34E-04	233	1301	NCOA3,ABCA1,ABL1,AOC1,TRARG1,CLTRN,CARD16,ADCY2,ADCY3,ADCY5,ADCY7,ADD1,ADH5,FZD4,GRK2,OSBPL7,WDR35,KLF11,TEAD2,ADCY4,PIK3R3,GRAMD1A,APOB,AR,RHOB,ARRB1,ARRB2,KLF7,USO1,PDE8B,LAMTOR3,HAMP,ATP2B4,UBR1,ATP6V1B2,ATP6V1C1,ATP6V0B,BGLAP,BMP6,CDK5R1,KLF5,CASTOR3,CACNA1E,CACNA2D1,TRIM41,CASP1,CASP9,USP8,AIFM1,ARHGEF2,CDK4,RAB11B,KLF4,CHRM2,CHRM3,HMGN3,GLP2R,CACTIN,RRAGD,RECQL5,LRP5L,MAP4K4,LY86,COL1A1,COL1A2,COL3A1,COL6A1,MPC2,COL16A1,GDF15,NR1D1,KANK2,SOGA1,HDAC9,CYP24A1,SIRPA,AKR1C1,STAP1,CCS,DNM2,DRD1,HDAC5,BCL2L11,ACTR2,SIDT2,NAMPT,AKAP9,SIGIRR,EIF4E,PLEKHA1,CALCRL,MTDH,EPRS1,ESD,ETS1,TCIRG1,KLF2,PTK2B,RAPGEF3,FOXC2,LARS1,SORBS1,ANKRD1,FOXP1,NDOR1,PPARGC1A,GJB3,PRDX3,GNA11,GNAI3,GNAL,GNB1,FFAR2,KLHL22,GUCY1B1,MAP4K1,H2AZ1,PDCD10,HK3,HMGCR,HNRNPK,UGT3A2,HSF1,ID1,ID3,IFNG,RGS8,IGF2,C1QTNF12,IL6,IL15,INHBB,CXCL10,IRF3,ITPR1,ITPR3,KCNE1,KIF5B,LRP1,LY6E,MAP1B,MAX,MEN1,FOXO4,MYD88,MYO1C,NFATC4,NFE2L2,NME1,VWA2,NTRK3,NUCB2,OXCT1,PRDX1,PALM,PAX2,PCK2,PDGFB,PDGFRB,SGMS1,PIK3C2A,XRN1,SLC2A8,TNIP2,MIR140,MIR16-2,PRKAA1,PRKAR1A,PRKAR2B,PRKCE,PRKCI,MAPK7,MAPK13,GRAMD1C,TRPM4,PTGDR,PTK6,PTPN2,PTPRF,CASTOR1,PXN,CDC73,

						RANGAP1,RAP1B,RARA,RARG,RARRES2,ANO1,UPF1,ZFAND1,RORA,RYR1,SLC30A10,SHC1,SIPA1,SLC6A4,VPS35,CPEB4,LMBRD1,SP1,SRC,HTR3E,STAT6,STC1,VAMP2,TAF1,PDGFC,TBX1,TBXA2R,SZT2,NCSTN,TGFB1,RHOQ,KLF10,AGRN,TP53,TRPM2,TSC2,LY96,COLEC12,UBTF,UCHL3,SMYD3,CYP26B1,P2RY12,WNT5A,WNT9B,LPIN3,SLC26A6,SPHK2,ENY2,PDXP,NADK
GO:0045785	positive regulation of cell adhesion	5.49E-08	5.75E-04	101	465	ABL1,ADA,PREX1,CHRD,ZFHX3,TNFSF14,TNFSF9,AZU1,SART1,CD27,CD47,CDC42,CDK6,MAP4K4,PRKD2,COL8A1,COL16A1,MAP3K8,PPM1F,SOCS5,CYLD,PIEZO1,SIRPA,DBN1,DMP1,DNM2,PTPRU,EFNB1,EFNB2,HSD17B12,ERBB2,FSTL3,ETS1,EMILIN2,CITED2,PTK2B,BTN2A2,VAV3,FOXF1,FOXC2,EGFLAM,CD244,GATA3,GCNT2,GLI2,GLI3,EMILIN1,HLA-DMB,HSPD1,IBSP,IFNG,IGF2,IHH,IL4,IL4R,IL6,IL6R,IL15,ITGA3,CD46,AFDN,UNC13D,CCDC88B,NID1,NODAL,PNP,NPY2R,PCSK5,PDCD1,PLD2,APBB1IP,EPB41L4B,PODXL,PRKCE,PTPRC,NDNF,RARA,MMRN2,CCL19,XCL1,SDC4,IFT74,SLC4A1,LIMS2,SPTA1,SRC,SMOC1,VSIR,CARMIL2,NFKBIZ,TFE3,TGFB1,TGM2,TNXB,TSC2,VAV1,VEGFA,P2RY12,WNT5A,DOCK8,ZMIZ1
vGO:0060349	bone morphogenesis	1.02E-07	1.06E-03	39	127	ALPL,ANXA2,SCUBE2,BMP6,SERPINH1,CBS,DHRS3,LRP5L,COL1A1,COL6A1,COL6A2,COL6A3,COL7A1,COMP,OSR2,CITED2,FGFR3,SCARA3,FOXC1,CARM1,GLG1,GLI3,HSPG2,IHH,LTBP3,MATN1,MEF2D,VWA2,RARA,RARG,CHSY1,SKI,STC1,PHOSPHO1,TGFB1,THBS3,COL14A1,CYP26B1,VWA1
GO:0001501	skeletal system development	1.27E-07	1.33E-03	119	582	ACVRL1,DSCAML1,ALPL,WDR48,ANXA2,SCUBE2,DCHS1,CHRD,BGLAP,BMP1,BMP2,BMP6,PAPSS1,SERPINH1,CBS,CDH11,DHRS3,CHAD,CMKLR1,LRP5L,COL1A1,COL1A2,COL3A1,COL6A1,COL6A2,COL6A3,COL7A1,COL11A1,COMP,ADAMTS4,PKDCC,OSR2,CTSK,HDAC4,SLC38A10,DLG1,MED12,CLEC3A,WASF2,PLEKHA1,ENG,EP300,LRRC17,IRX5,SPNS2,EVC,CITED2,FGFR3,SCARA3,FOXC1,PRRX2,FOXC2,CARM1,WFIKKN1,FOXP1,SUFU,GAS1,GLG1,GLI2,GLI3,GNA11,ALX4,HOXD1,HSPG2,IGF2,IHH,SCIN,INHA,LTBP3,MAF,MUSTN1,MATN1,MDFI,MEF2D,MEN1,FGFRL1,NODAL,VWA2,NOTCH2,PRDX1,RFLNA,PBX1,PCSK5,PDGFRB,RANBP3L,PRELP,PTH1R,PTPRC,RARA,RARG,LRRK1,SPEF2,RYR1,CTC1,CHSY1,SKI,SNAI1,SOX11,SP1,SRC,STC1,SULF2,PHOSPHO1,NBEAL2,CCDC154,PDGFC,TBX1,TCOF1,TGFB1,THBS3,THPO,TP53,COL14A1,VEGFA,CYP26B1,WNT5A,WNT9B,VWA1,ZIC1
GO:0001503	ossification	1.46E-07	1.53E-03	96	444	ALPL,SCUBE2,DCHS1,CHRD,BGLAP,BMP1,BMP2,BMP6,CBS,CDH11,CDK6,CEBPD,DHRS3,PDLIM7,CLIC1,LRP5L,COL1A1,COL1A2,COL6A1,COL11A1,COMP,PKDCC,OSR2,CTSK,HDAC4,CYP24A1,MRC2,DMP1,HDAC5,CLEC3A,LRRC17,KREMEN1,FSTL3,TCIRG1,EXT2,ADGRV1,PTK2B,FGFR3,GPNMB,SUCO,FOXC1,FOXC2,MYBBP1A,HDAC7,SUFU,TOB2,GLI2,GLI3,HSPG2,IARS1,IBSP,ID1,ID3,ID4,IGF2,IGFBP3,IHH,IL6,IL6R,LRP4,LTBP3,SMAD6,MATN1,MEF2D,MEN1,MN1,NOTCH1,DDR2,RFLNA,PBX1,RANBP3L,TRPM4,PTH1R,RYR1,CLEC11A,ITGA11,CHSY1,SKI,SNAI1,SP7,SOX11,SP1,ZHX3,SMOC1,STC1,PHOSPHO1,BCAP29,CCDC154,TGFB1,THBS3,KLF10,HEY1,HIRA,VEGFA,WNT5A,CCDC47
GO:0097435	supramolecular fiber organization	2.48E-07	2.60E-03	141	727	ABL1,ADD1,TTC8,AEBP1,PREX1,ANXA2,APOE,RHOB,RHOG,ARHGAP6,ARRB1,PHACTR1,MYOM1,B2M,RAB11A,CDK5R1,CAPZA1,CAPZA2,CASQ1,SERPINH1,CD47,ARHGEF2,CDC42,CDH5,CFL2,MID1IP1,MYOZ1,LMOD1,COL1A1,COL1A2,COL3A1,COL5A1,COL11A1,COMP,ADAMTS2,KANK2,PPM1F,NEURL2,AIF1L,SIRPA,DBN1,DES,FCHSD2,VILL,DIAPH2,DLG1,DPYSL3,WASHC3,ACTR2,AKAP9,WASF2,SORBS3,EP300,F2RL1,PTK2B,FAT1,RAPGEF3,FOXC1,FOXC2,SORBS1,ANKRD1,FOXP1,INPP5J,SPIRE2,MYOZ2,GMFB,TMSB15A,KATNA1,EMILIN1,GSN,CDC42EP1,LDB3,FAM107A,KANK3,CORO6,PDCD10,SYNPO,HSPA1A,HSPA8,ID1,SHANK3,SCIN,KIF2A,KPNB1,KRT19,LCP1,LMX1B,ARHGAP12,ARPIN,LTBP2,MAP1B,MYOM3,FHOD1,MYH6,MYH11,MYO1C,CCDC88B,NF2,NRAP,DDR2,NUMA1,P4HA1,RFLNA,PDGFRB,PLOD2,PLS1,PACSIN1,EFEMP2,SH3KBP1,PRKAR1A,PRKCE,PRKCI,KLHL24,PXN,CCDC88C,MAP1S,ARHGAP28,SDC4,ARHGEF15,SPTA1,SPTAN1,SRC,NEDD1,RHOBTB2,CARMIL2,OBSL1,TESK1,RHOQ,TMOD1,TNXB,TPM2,SYNPO2,CD2AP,SH3BP1,COL14A1,MICAL1,WIPF1,FMN2,PDXP,NAV3,FCHSD1
GO:0030198	extracellular matrix organization	3.79E-07	3.97E-03	87	399	ABL1,ADAM10,AEBP1,ANXA2,ITGA10,ITGA8,BMP1,SERPINH1,CD47,COL1A1,COL1A2,COL3A1,COL5A1,COL6A1,COL6A2,COL6A3,COL7A1,COL8A1,COL9A3,COL11A1,COL15A1,COL16A1,COMP,ADAMTS4,ADAMTS2,OLFML2B,COL23A1,SH3PXD2A,SCUBE3,CTSK,CTSL,CTSV,DMP1,HSD17B12,ENG,ETS1,LAMC3,SCARA3,FOXF1,FOXF2,FOXC1,FOXC2,EGFLAM,ADTRP,EMILIN1,GSN,HAS1,RIC8A,HSPG2,IBSP,IHH,IL6,ITGA3,ITGA9,LAMA2,LCP1,LMX1B,LRP1,MATN1,MELTF,MMP8,MYH11,MYO1E,NID1,NOTCH1,DDR2,P4HA1,FURIN,PDGFB,COL22A1,PLOD2,EFEMP2,OLFML2A,NDNF,ITGA11,SCUBE1,SMOC1,SULF2,CARMIL2,TGFB1,TIMP2,AGRN,TNXB,COL14A1,VWF,VWA1,ADAM12
GO:0030155	regulation of cell adhesion	3.84E-07	4.03E-03	148	777	ABL1,ACVRL1,ADA,ADAM10,DSCAML1,FZD4,MAD1L1,PREX1,ARHGAP6,ARHGDIA,CHRD,ZFHX3,TNFSF14,TNFSF9,AZU1,BMP2,BMP6,SART1,CD9,CD27,CD47,CDC42,CDK6,CYTH2,CYTH1,KLF4,MAP4K4,COL1A1,ONECUT2,PRKD2,COL8A1,COL16A1,MAP3K8,MINK1,CCM2L,PPM1F,SOCS5,CYLD,RIPOR2,RC3H1,PIEZO1,SIRPA,DBN1,DLG1,DMP1,DNM2,ITCH,PTPRU,EFNB1,EFNB2,HSD17B12,B4GALNT2,ERBB2,FSTL3,ETS1,EMILIN2,CITED2,PTK2B,BTN2A2,VAV3,GPNMB,FOXF1,FOXC2,EGFLAM,CD244,LRRC32,GATA3,GCNT2,GLI2,GLI3,ADTRP,EMILIN1,GSN,FAM107A,HLA-DMB,HSPD1,IBSP,IFNG,IGF2,IHH,IL4,IL4R,IL6,IL6R,IL15,ITGA3,LAMA2,LRP1,DACT2,CD46,MEN1,MELTF,AFDN,MYO1F,NEXMIF,FXYD5,UNC13D,CCDC88B,NF2,NID1,NODAL,NOTCH1,PNP,NPY2R,DDR2,PCSK5,PDCD1,PLD2,PLXNA1,APBB1IP,PML,EPB41L4B,PODXL,PRKAR1A,PRKCE,MAPK7,PTPN2,PTPRC,NDNF,RARA,MMRN2,CCL19,XCL1,SDC4,PLXNA3,IFT74,C2CD4A,SIPA1, SLC4A1,LIMS2,SPTA1,SRC,PLXND1,SMOC1,VSIR,CARMIL2,NFKBIZ,TFE3,TGFB1,TGM2,TNXB,TSC2,VAV1,VEGFA,P2RY12,WNT5A,DOCK8,ZMIZ1
GO:0032989	cellular component morphogenesis	4.01E-07	4.20E-03	227	1297	ABL1,LRTM1,ADAM10,FMNL2,DSCAML1,ADD1,TBC1D24,FZD4,TTC8,CGN,STK24,PREX1,FGD2,CNTNAP1,ITGA8,APOE,AR,THOC5,RHOB,RHOG,ARHGDIA,KLF7,PHACTR1,MYOM1,RAB11A,BAX,NRP2,CDK5R1,CAMK2B,CASP9,CASQ1,FMNL3,CD9,MAP3K13,ARHGEF2,CDC42,CDH5,CDH6,CDH11,ZMYM3,CDH17,RPS6KA5,NUMBL,PDLIM7,CFL2,TAOK2,MYOZ1,LMOD1,NTN1,MAP4K4,COL6A1,COL6A2,COL6A3,COL7A1,MINK1,COL23A1,CNTNAP2,NEUROG3,CUX1,SART3,RIPOR2,NEURL2,DBN1,CUL7,FIG4,DLG1,DNM2,ACTR2,SIDT2,EFNB1,EFNB2,WASF2,PLEKHO1,EP300,EPB41L2,ERBB2,ETV4,LAMC3,NRN1,KLF2,PTK2B,DACT1,FAT1,FGFR3,SCARA3,SEMA6C,DPYSL4,NPTN,GNA13,ANKRD1,FOXP1,EGFLAM,GAS1,GATA3,MYOZ2,GLI2,GLI3,BHLHE22,GNA12,GOLGA4,VAX1,CDC42EP1,LDB3,KLK8,PACSIN2,HNRNPK,HSPG2,ID1,SHANK3,IFRD1,WTIP,IHH,IL6,KRT19,LAMA2,LRP1,LRP4,ARPIN,MAP1B,MATN1,DACT2,MELTF,MKLN1,PDZD8,AFDN,MOV10,MYOM3,C1GALT1C1,BCL9L,MYH6,MYH11,MYO9B,FGFRL1,UNC13D,NEO1,NFATC4,FGD5,NODAL,VWA2,NOTCH1,NOTCH4,CNTN4,NRAP,NTRK3,PALM,PAX2,PDGFRB,PNPT1,TFCP2L1,SEMA5B,PIK3CD,PLXNA1,ROBO4,PACSIN1,SH3KBP1,TMEM106B,PRKAR1A,MAPK7,FBLIM1,SEMA4C,PTPRF,PTPRS,SSH3,NECTIN1,NECTIN2,PXN,RNF6,MAP1S,EIF2AK4,SCN1B,PLXNA3,SHC1,NTNG1,NLGN1,PRPF40A,BTBD3,LIMS2,RADIL,DOK4,SLIT3,PARVA,FGD6,MYO16,ARHGAP15,SPTA1,SPTAN1,SRC,NFASC,SSBP1,PLXND1,LARP4,PARVG,SS18,ST14,STC1,NBEAL2,RHOBTB2,SCFD1,ROBO3,SZT2,JADE2,OBSL1,GRIP1,RHOQ,ARAP3,TMF1,TMOD1,PARD3,AGRN,TSC2,COL14A1,CCDC136,VEGFA,VLDLR,WNT5A,RTN4RL1,VWA1,AKIP1,YWHAH,SEMA3G,UNC93B1,FGD3,FZD3,RTN4,SLITRK4,THOC2,JMJD1C
GO:0060351	cartilage development involved in	4.14E-07	4.34E-03	22	55	ANXA2,SCUBE2,SERPINH1,CBS,COL1A1,COL6A1,COL6A2,COL6A3,COL7A1,COMP,SCARA3,CARM1,HSPG2,IHH,MATN1,VWA2,RARA,RARG,STC1,THBS3,COL14A1,VWA1

	endochondral bone morphogenesis					
GO:0060350	endochondral bone morphogenesis	5.73E-07	6.01E-03	28	82	ALPL,ANXA2,SCUBE2,BMP6,SERPINH1,CBS,COL1A1,COL6A1,COL6A2,COL6A3,COL7A1,COMP,FGFR3,SCARA3,FOXC1,CARM1,HSPG2,IHH,MATN1,MEF2D,VWA2,RARA,RARG,STC1,PHOSPHO1,THBS3,COL14A1,VWA1
GO:0003416	endochondral bone growth	6.35E-07	6.65E-03	20	48	ANXA2,COL6A1,COL6A2,COL6A3,COL7A1,COMP,EVC,FGFR3,SCARA3,CARM1,MATN1,VWA2,DDR2,BNC2,RARA,RARG,STC1,THBS3,COL14A1,VWA1
GO:0009887	animal organ morphogenesis	1.17E-06	1.23E-02	216	1241	NCOA3,ABL1,ACTG2,ACVRL1,ARID1A,DSCAML1,FZD4,FZD6,TTC8,SLC24A4,AP2A1,AP2A2,ALPL,WDR48,TEAD2,TSHZ3,ANXA2,ITGA8,AQP3,AR,ARRB2,SCUBE2,DCHS1,DNAH11,MYOM1,BAX,BGLAP,NRP2,BMP1,BMP2,BMP6,CASP6,SERPINH1,CBS,KCNQ4,CDC42,DHRS3,CHAD,NTN1,LRP5L,COL1A1,COL1A2,COL3A1,ONECUT2,COL5A1,COL6A1,COL6A2,COL6A3,COL7A1,COL8A1,COL11A1,COMP,OSR2,SOSTDC1,ATF2,CCM2L,CTSV,RIPOR2,DLG1,MAFB,DLX3,DMP1,MED12,CDON,DGCR2,BCL2L11,HEYL,EFNB2,PLEKHA1,ENG,EP300,IRX5,ETV4,TCIRG1,LAMC3,CITED2,DACT1,FAT1,FGFR3,FHL1,SCARA3,FOXF1,FOXF2,FOXC1,PRRX2,FOXC2,CARM1,SIRT6,ANKRD1,EGFLAM,SUFU,GAA,GAK,GAS1,GATA2,GATA3,GATA6,PPP1R13L,GLG1,GLI2,GLI3,BHLHE22,TMED2,ALX4,HSPG2,ID1,ID3,ID4,SHANK3,IFNG,IGF2,ACP4,IHH,IL6,ITPR1,LAMA2,LFNG,LRP4,LTBP3,LY6E,SMAD6,MATN1,MDFI,MEF2D,MEN1,AFDN,MYOM3,MYH6,MYL3,MYLK,MYO6,FGFRL1,NF2,NFIC,NODAL,VWA2,NOTCH1,NOTCH2,NPY2R,NPY5R,PAX2,RFLNA,PBX1,PDGFB,PDGFRB,SOX18,PGF,PLXNA1,PML,ARHGEF19,IRX3,IRX6,PPP2R3A,PRKAR1A,PRKCI,FAM20A,PSMA2,PSMA3,PSMB1,PSMC6,PSMD3,PSMD12,NECTIN1,RARA,RARG,SPEF2,RXRA,RYR1,NTNG1,CHSY1,SKI,LIMS2,SLC6A4,SLC12A2,SLIT3,PARVA,SP6,SMARCD3,KDM2A,SNAI1,SOX11,SP1,SRC,PLXND1,STAT6,STC1,SULF2,PHOSPHO1,CCDC154,PDGFC,TBX1,TBX2,TCF21,OBSL1,TGFB1,TGM2,THBS3,HEY1,TLE1,TLE2,TLE3,HEY2,AGRN,TP53,COL14A1,USH2A,VEGFA,CYP26B1,WNT5A,WNT9B,VWA1,ZIC1,ZNF22,FZD3,RTN4,ZMIZ1
GO:0007265	Ras protein signal transduction	1.29E-06	1.35E-02	98	477	ABCA1,LZTR1,ABL1,ADRB1,PREX1,FGD2,DGKZ,APOE,MADD,RHOB,RHOG,ARHGAP6,ARHGDIA,ARRB1,RAB40C,CTNNAL1,RAB11A,CBL,USP8,ARHGEF1,DGKI,ARHGEF2,CDC42,RAB11B,RASGRP4,CYTH2,CYTH1,SHC2,NTN1,MAP4K4,COL1A2,COL3A1,RAB3D,KANK2,RIPOR2,ARHGEF17,ELMO1,FARP2,G3BP2,DENND4B,DNM2,TRIM28,LPAR6,WASF2,RASGRP2,SPRY3,ERBB2,DNAJC27,F2RL1,F2RL2,RAB6C,RAPGEF3,VAV3,HACD3,GNA13,CYTH4,ARPP19,GNA12,GNB1,MAPRE2,GPR17,CDC42EP1,PDCD10,RERG,ITGA3,KPNB1,MYO9B,RAB4B,FGD5,NOTCH1,NOTCH2,PDGFRB,ARHGEF19,MAPK11,RIPOR1,RAB4A,RAP1B,TAX1BP3,RASA3,SHC1,ARHGEF15,RAB18,ARHGEF40,FGD6,RHOBTB2,RAB17,RHOQ,ARAP3,TIMP2,TP53,NUP62,VAV1,IFT22,FGD3,RTN4,RASGEF1A,SHOC2,SPATA13
GO:0072358	cardiovascular system development	1.29E-06	1.36E-02	164	897	ABL1,ACVRL1,ARID1A,ADAM10,ADD1,FZD4,JCAD,TEAD2,ANPEP,ANXA2,PIK3R3,APOB,APOE,RHOB,ADGRB2,BAX,NRP2,KLF5,KRIT1,FMNL3,CDC42,CDH5,AIMP1,CHM,KLF4,LRP5L,COL1A1,COL1A2,COL3A1,COL5A1,PRKD2,COL8A1,COL15A1,COMP,ADGRA2,ATF2,COL23A1,CSPG4,PPP1R16B,HDAC9,ISM1,CUL7,DLX3,DNM2,SLC12A6,HDAC5,E2F2,EFNB2,WASF2,CALCRL,ENG,MTDH,ERBB2,SPEG,ETS1,WARS2,KLF2,CITED2,PTK2B,RAPGEF3,VAV3,GPNMB,AMOTL2,FOXF1,FOXC1,PRRX2,FOXC2,FLT4,SIRT6,HDAC7,GNA13,FOXP1,SUFU,GATA2,GATA6,GJA4,GLI3,TMED2,GRN,ADTRP,EMILIN1,PDCD10,RIC8A,HSPB1,HSPG2,ID1,IFNG,IGF2,HSPB6,IHH,IL6,IL6R,CXCL10,LRP1,SMAD6,MCAM,MAP3K3,MIR2355,FOXO4,MYLK,MYO1E,NDP,NFATC4,NFE2L2,NODAL,NOTCH1,NOTCH2,NOTCH3,NOTCH4,PCSK5,PDGFB,PDGFRB,SOX18,CARD10,PGF,PIK3C2A,PDCL3,PLCD1,PML,ROBO4,EFEMP2,MIR140,MIR16-2,CLEC14A,MAPK7,MIR34C,PTGIS,PXN,NDNF,TMEM204,DYNC2H1,KIF7,MMRN2,RORA,RXRA,STAB2,NAA15,SGCB,SHC1,HIF1AN,ARHGEF15,PARVA,SP1,PLXND1,STAB1,TBX1,TBX2,TBXA2R,TCF21,HIF3A,NR2F2,TGFB1,TIE1,HEY1,HEY2,TNFAIP2,VEGFA,WNT5A,SPHK2,APOLD1,RTN4,ZMIZ1,ADAM12,NPRL3
GO:0001944	vasculature development	1.30E-06	1.36E-02	162	884	ABL1,ACVRL1,ARID1A,ADAM10,ADD1,FZD4,JCAD,TEAD2,ANPEP,ANXA2,PIK3R3,APOB,APOE,RHOB,ADGRB2,BAX,NRP2,KLF5,KRIT1,FMNL3,CDC42,CDH5,AIMP1,CHM,KLF4,LRP5L,COL1A1,COL1A2,COL3A1,COL5A1,PRKD2,COL8A1,COL15A1,COMP,ADGRA2,ATF2,COL23A1,CSPG4,PPP1R16B,HDAC9,ISM1,CUL7,DLX3,DNM2,SLC12A6,HDAC5,E2F2,EFNB2,WASF2,CALCRL,ENG,MTDH,ERBB2,ETS1,WARS2,KLF2,CITED2,PTK2B,RAPGEF3,VAV3,GPNMB,AMOTL2,FOXF1,FOXC1,PRRX2,FOXC2,FLT4,SIRT6,HDAC7,GNA13,SUFU,GATA2,GATA6,GJA4,GLI3,TMED2,GRN,ADTRP,EMILIN1,PDCD10,RIC8A,HSPB1,HSPG2,ID1,IFNG,IGF2,HSPB6,IHH,IL6,IL6R,CXCL10,LRP1,SMAD6,MCAM,MAP3K3,MIR2355,FOXO4,MYLK,MYO1E,NDP,NFATC4,NFE2L2,NODAL,NOTCH1,NOTCH2,NOTCH3,NOTCH4,PCSK5,PDGFB,PDGFRB,SOX18,CARD10,PGF,PIK3C2A,PDCL3,PLCD1,PML,ROBO4,EFEMP2,MIR140,MIR16-2,CLEC14A,MAPK7,MIR34C,PTGIS,PXN,NDNF,TMEM204,DYNC2H1,KIF7,MMRN2,RORA,RXRA,STAB2,NAA15,SGCB,SHC1,HIF1AN,ARHGEF15,PARVA,SP1,PLXND1,STAB1,TBX1,TBX2,TBXA2R,TCF21,HIF3A,NR2F2,TGFB1,TIE1,HEY1,HEY2,TNFAIP2,VEGFA,WNT5A,SPHK2,APOLD1,RTN4,ZMIZ1,ADAM12,NPRL3
GO:0007507	heart development	1.72E-06	1.80E-02	133	700	ACADM,ACVRL1,ARID1A,ADRB1,GRK2,TEAD2,ANXA2,LMO4,ARRB2,DCHS1,HAMP,DNAH11,MYOM1,NRP2,HDAC3,BMP2,KLF5,CACNA1C,CAD,DAW1,CDC42,DHRS3,PDLIM7,COL3A1,COL5A1,COL11A1,ATF2,FOXP4,CCM2L,HDAC9,MAML1,TMEM65,MED12,DNM2,HDAC5,ECE1,HEYL,EFNB2,CALCRL,ENG,EP300,ERBB2,ERBB4,SPEG,CITED2,FOXF1,FOXC1,FOXC2,ZDHHC16,SIRT6,ANKRD1,FOXP1,SUFU,G6PD,GAA,GATA3,GATA6,PPP1R13L,GLI2,GLI3,TMED2,GNA11,EMILIN1,LDB3,HSPG2,ID1,ID3,IHH,ITGA3,LRP1,LY6E,SMAD6,MBD1,MEF2D,MYOM3,MYH6,MYH11,MYL3,RIPPLY3,FGFRL1,NFATC4,NODAL,NOTCH1,NOTCH2,NPY2R,NPY5R,NRAP,NTRK3,OXCT1,OXTR,PCSK5,PDGFB,PDGFRB,SOX18,PRICKLE4,PRKAR1A,MAPK11,RARA,DYNC2H1,KIF7,RXRA,RYR1,SGCB,IFT74,SGCG,SHC1,SCUBE1,SLIT3,PARVA,SMARCD3,KDM2A,SNAI1,ADAP2,SOX11,TAB2,PLXND1,SMG9,PDLIM2,TBX1,TBX2,OBSL1,TGFB1,HEY1,HEY2,TP53,TSC2,COL14A1,VEGFA,WNT5A,RTN4,JPH2,ZMIZ1,NPRL3
GO:0051241	negative regulation of multicellular organismal process	1.80E-06	1.88E-02	253	1500	CARD16,ACVRL1,ADA,H4C9,ADCY7,ADRA1D,ADRA2C,ADRB1,GRK2,MAVS,ARRDC3,ANPEP,ANXA2,APOA2,APOE,THOC5,MADD,KHSRP,ARHGDIA,ARRB1,ARRB2,KLF7,CHRD,HES4,HAMP,ATP2B4,B2M,ADGRB2,HDAC3,BMP2,CDK5R1,KLF5,TAX1BP1,SERPING1,CACNB3,CTDSP1,KRIT1,NMI,CD9,ARHGEF2,CDK6,RPS6KA5,CYTH2,CHAD,KLF4,CACTIN,NTN1,CMKLR1,MAP4K4,COL3A1,COL5A1,GDF15,SOSTDC1,NR1D1,ATF2,CRY2,ZBTB46,FOXP4,SOCS5,CSPG4,PHF14,CTSK,RAB11FIP3,CYLD,HDAC9,HDAC4,RC3H1,ISM1,SIRPA,DBN1,STAP1,FIG4,MAFB,THOC1,DPYSL3,DRD1,ITCH,HDAC5,TSC22D3,E2F2,EFNB2,SIGIRR,EIF4E,CALCRL,LRRC17,ERBB2,ERBB4,KREMEN1,FSTL3,ETV4,MRVI1,F2RL1,TLR8,KLF2,CITED2,PTK2B,BTN2A2,FGFR3,GPNMB,FOXC1,FOXC2,CARM1,SEMA6C,RNF135,HDAC7,NPTN,KLF13,FOXP1,ASB1,SUFU,G6PD,INPP5J,TOB2,GAK,LRRC32,GATA2,GATA3,GATA6,HPGDS,GLI2,GLI3,STK39,VAX1,LILRB3,GRN,ADTRP,KATNA1,EMILIN1,KLK8,PDCD10,HLA-F,HMGCR,HNRNPK,HSF1,HSPA9,HSPG2,ID1,ID3,ID4,IFNG,IFRD1,ACP4,IHH,IL4,IL4R,IL6,IL15,INHA,INHBB,CXCL10,IRF3,KCNC4,KCNMA1,LFNG,LRP1,LRP4,LTBP3,SMAD6,MBD1,MCC,MEN1,MIR2355,FOXO4,MMP8,TBK1,NEXMIF,NEO1,NFATC4,NFE2L2,NME1,NODAL,NOTCH1,NOTCH3,CNTN4,NPY2R,NTRK3,OXTR,FURIN,PAX2,RFLNA,PBX1,PDCD1,PDGFB,CARD10,SEMA5B,PLCL1,PML,DHX58,IRX3,MIR16-

						2,RANBP3L,MIR181B2,MAPK11,MIR34C,TRPM4,SEMA4C,PTPN2,PTPRC,RNF125,PTPRS,CDC73,RARA,RARG,RNF6,TRIM62,MMRN2,EIF2AK4,RPS19,XCL1,PLXNA3,SHC1,CHSY1,NLGN1,SKI,ARHGEF15,LIMS2,SLC6A4,GPATCH3,SNAI1,SOX11,ZNF423,VSIR,STC1,STAB1,TBX2,TBXA2R,NR2F1,NR2F2,TFE3,TGFB1,GPR161,TIE1,HEY1,TLE3,HEY2,DAAM2,TP53,CD2AP,TSC2,TNFRSF4,UBA7,VEGFA,WNT5A,WNT9B,RTN4RL1,YWHAH,SEMA3G,TRIM11,IL1R2,NAV3,FZD3,RTN4,THOC2
GO:0035295	tube development	1.87E-06	1.96E-02	220	1276	NCOA3,ABL1,ACAT1,ACVRL1,ARID1A,ADA,ADD1,FZD4,FZD6,TTC8,WDR48,JCAD,TEAD2,TSHZ3,ANPEP,ANXA2,PIK3R3,APOB,APOE,LMO4,AR,RHOB,ASAH1,DCHS1,CHRD,ADGRB2,BAX,NRP2,BMP2,KLF5,KRIT1,FMNL3,CDC42,CDH5,CRLF1,AIMP1,KLF4,NTN1,LRP5L,COL3A1,PRKD2,COL8A1,COL15A1,COMP,ADAMTS2,PKDCC,SOSTDC1,ADGRA2,ATF2,COL23A1,FOXP4,CSPG4,PPP1R16B,PHF14,CTSV,CUX1,HDAC9,CYP1A1,ISM1,CUL7,DLG1,MED12,SLC12A6,HDAC5,BCL2L11,E2F2,EDN2,HEYL,EFNB2,WASF2,EIF4E,CALCRL,ENG,MTDH,EP300,ERBB2,FSTL3,ETS1,ETV4,WARS2,KLF2,CITED2,PTK2B,DACT1,RAPGEF3,VAV3,FGFR3,GPNMB,AMOTL2,FOXF1,FOXF2,FOXC1,PRRX2,FOXC2,FLT4,SIRT6,HDAC7,GNA13,FOXP1,SUFU,GAK,GAS1,GATA2,GATA3,GATA6,GJB5,MAN1A2,GLI2,GLI3,TMED2,CECR2,GRN,ADTRP,EMILIN1,ATOH8,PDCD10,ALX4,HSPB1,HSPG2,ID1,SHANK3,IGF2,HSPB6,IHH,IL6,CXCL10,ITGA3,LRP1,LTBP3,INKA1,SMAD6,DACT2,MCAM,MAP3K3,MIR2355,FOXO4,MYLK,MYO1E,NFATC4,NFE2L2,NODAL,NOTCH1,NOTCH2,NOTCH3,NOTCH4,NUMA1,OXTR,PAX2,PBX1,PCSK5,PDGFB,PDGFRB,SOX18,CARD10,PGF,PIK3C2A,PDCL3,PLCD1,PML,ROBO4,PODXL,IRX3,MIR16-2,CLEC14A,MAPK7,MIR34C,PTGIS,PTK6,SEMA4C,PXN,NDNF,RARA,RARG,RARRES2,MMRN2,RORA,MEG3,RXRA,SDC4,STAB2,NAA15,SHC1,SKI,HIF1AN,SLC12A2,PARVA,KDM2A,SOX11,SP1,SRC,PLXND1,ST14,STAB1,PDGFC,TBX1,TBX2,TBXA2R,TCF21,HIF3A,NR2F2,TGFB1,GPR161,TGM2,TIE1,HEY1,HEY2,TNFAIP2,TSC2,TNS3,VEGFA,WNT5A,WNT9B,APOLD1,FZD3,RTN4,ZMIZ1,ADAM12,JMJD1C,NPRL3,RCN3

Supplemental Table 1. ToppGene Analysis showing the top 30 enriched Biological Processes

Table S2. MSD immunoassay analysis of CP lysates from ALS and non-neurological disease controls.

Sample	Group	MMP-1	MMP-3	MMP-9	Fractalkin	MCP-1	M-CSF	VEGF-A	CRP	SAA	VCAM-1	ICAM-1	E-Selectin	P-Selectin	TM	IL-1B	IL-6	IL-8	IFN-g
SALS6	ALS	1.27	2.35	239.64	47.86	2.17	0.89	2.79	6876.67	9697.98	346.42	594.124	0.0219	0.2773	0.641	0.0132	0.324	1.024	0.0048
SALS1	ALS	3.17	1.94	248	80.86	5.46	0.75	4.32	448.58	3654.99	599.81	845.795	0.03	0.6118	1.181	0.0316	0.677	0.371	0.0094
SALS2	ALS	8.7	5.13	955.41	51.92	1.04	0.66	1.53	108859	223357	409.01	724.391	0.0551	1.0968	0.893	0.0127	0.118	0.244	0.0145
SALS3	ALS	0.73	1.73	140.88	54.31	6.79	0.26	1.39	77.49	250.5	285.52	370.023	0.0155	0.3533	0.503	0.0114	0.219	1.209	0.0050
SALS7	ALS	1.29	8.57	320.6	71.5	15.25	0.57	1.23	6286.23	50980.5	200.13	877.151	0.0212	0.3776	0.945	0.0749	0.554	0.679	0.0185
SALS4	ALS	3.76	12.33	185.28	47.18	1.89	0.92	2.26	30532.8	44906.6	151.42	510.214	0.0295	0.6128	1.255	0.0239	0.177	0.293	0.0128
SALS8	ALS	0.3	2.37	210.48	29.63	0.72	0.25	0.54	2056.99	810.72	104.02	241.513	0.0244	0.2865	0.328	0.0050	0.029	0.080	0.0064
SALS9	ALS	0.41	2.13	163.75	67.58	3.43	1.11	3.66	580	567.87	141.07	424.797	0.0528	0.3129	1.066	0.0246	0.185	0.264	0.0095
SALS10	ALS	1.21	2.5	837.05	65.83	3.14	0.64	1.77	6688.08	19191.6	265.68	551.453	0.0238	0.4994	0.848	0.0109	0.099	0.208	0.0158
SALS17	ALS	4.6	1.74	917.5	66.71	5.83	0.94	2.2	47095.4	150407	276.89	696.683	0.0453	0.6667	0.657	0.0207	0.229	0.166	0.0213
SALS5	ALS	1.41	3.59	1020.27	53.56	19.58	1	0.95	4021.01	33509.9	209.73	795.12	0.0587	0.4017	1.060	0.0312	0.125	0.411	0.0112
C9-ALS4	ALS	1.09	1.16	358.27	81.32	3.21	0.75	1.82	2449.78	3271.49	176.39	593.54	0.0206	0.5929	0.771	0.0110	0.087	0.113	0.0095
SALS 33	ALS	0.3	1.56	220.82	43.87	1.46	0.5	1.35	695.37	1870.17	562.51	682.141	0.0145	0.2649	0.574	0.0044	0.014	0.087	0.0057
C9-ALS1	ALS	0.16	2.27	410.28	96.98	5.51	0.88	2.43	2393.92	1640.47	279.91	717.818	0.044	0.2979	0.842	0.0129	0.346	0.315	0.0138
C9-ALS2	ALS	0.67	4.07	283	48.01	2.17	0.64	1.12	1465.44	2366.57	215.58	444.754	0.0215	0.476	1.021	0.0157	0.164	0.379	0.0084
C9-ALS3	ALS	0.56	5.97	273.88	46.08	5.36	0.99	2.35	4339.15	3457.23	269.76	766.906	0.0263	0.3231	0.792	0.0617	0.335	0.928	0.0033
SALS 47	ALS	0.40	1.76	295.24	92.52	0.86	0.81	2.14	3787.57	3359.31	586.80	1174.98	0.0243	0.2815	0.785	0.0078	0.065	0.143	0.0101
SALS 32	ALS	-0.09	6	890.62	71.84	7.08	2.07	5.54	3392.34	9381.08	668.98	2546.5	0.0209	0.2968	1.444	0.1020	0.523	2.188	0.0546
SALS11	ALS	21.44	5.6	563.42	118.87	3.71	1.46	1.1	2665.53	5080.75	899.26	2241.11	0.0292	0.9858	2.873	0.0231	0.145	0.468	0.0484
fALS 1	ALS	4.22	7.62	382.29	160.98	4.6	1.8	1.18	2612.46	6514.45	376.48	1142.48	0.0284	0.4902	1.159	0.0234	0.151	0.710	0.0029
SALS 27	ALS	7.72	11.04	1171.64	64.54	11.09	1.77	0.67	2745.58	14988	1205.47	2430.97	0.0985	0.5094	1.936	0.0765	1.516	1.676	0.0111
SALS 28	ALS	25.28	3.25	1530.93	56.21	5.12	1.96	2.89	2992.78	16991	511.89	2056.59	0.0225	0.3884	1.612	0.0762	0.517	1.780	0.0145
SALS 29	ALS	4.48	3.38	1135.21	197.4	2.57	1.69	1.93	2620.05	5609.45	876.05	2132.28	0.0309	0.3443	1.245	0.0243	0.038	0.253	0.0182
SALS 30	ALS	3.01	11.72	595.03	150.77	7.75	1.8	3.45	2622.85	6834.66	426.74	1571.09	0.0197	0.5309	1.596	0.2728	0.306	1.028	0.0151
SALS 31	ALS	0.97	11.77	357.29	102.86	16.56	1.94	2.12	2595.58	4805.81	228.6	1593.71	0.016	0.0652	0.754	0.0552	0.210	1.693	0.0312
SALS23	ALS	0.76	0.55	145.91	46.6	1.28	0.24	0.35	545.38	125.23	213.46	398.981	0.019	0.1917	0.253	0.0111	0.024	0.173	0.0012
SALS 26	ALS	1.37	1.44	288.79	56.1	2.92	0.53	0.34	8606.35	19785	223.3	615.626	0.0189	0.237	0.217	0.0189	0.150	0.294	0.0009
C9-ALS8	ALS	1.97	1.11	58.22	34.51	3.13	0.68	0.54	10448.4	18565.9	200.82	694.645	0.0063	0.085	0.118	0.0642	0.343	0.666	0.0007
SALS 34	ALS	2.69	2.68	241.3	39.5	0.86	0.36	0.45	5777.42	18387.6	207.18	437.407	0.011	0.2302	0.248	0.0133	0.016	0.169	0.0012
SALS 41	ALS	0.59	2.01	115.97	35	2.43	0.37	0.41	7776.02	18791.8	198.79	301.904	0.0051	0.1224	0.166	0.0136	0.103	0.449	0.0012
SALS 40	ALS	0.13	0.64	116.95	34.6	1.08	0.32	0.17	122.39	308.61	359.41	603.991	0.0064	0.1856	0.245	0.0107	0.012	0.172	0.0004
SALS 42	ALS	0.67	2.07	109.15	72.83	1.53	0.45	0.54	78.21	153.48	283.77	415.043	0.0104	0.2382	0.317	0.0098	0.008	0.136	0.0002
SALS 36	ALS	1.21	0.28	146.87	24.28	0.96	0.19	0.25	630.68	512.34	141.07	325.051	0.0016	0.142	0.193	0.0213	0.044	0.163	0.0031
SALS 37	ALS	0.09	0.25	89.88	16.42	0.65	0.22	1.74	70.56	3088.76	31.64	110.134	0.0005	0.0123	0.054	0.0078	0.007	0.060	0.0012
SALS 46	ALS	0.76	0.66	92.24	24.14	0.62	0.28	0.24	2254.64	11529	211.09	585.035	0.0085	0.1404	0.261	0.0258	0.010	0.247	0.0014
SALS 44	ALS	7.24	1.81	496.93	50.04	4.44	0.51	1.17	12931.8	18215.2	268.62	974.862	0.0456	0.2038	0.237	0.0213	0.675	0.645	0.0035
C9-ALS9	ALS	0.3	0.66	138.16	31.84	5.52	0.57	1.27	997.91	998.76	173.69	681.454	0.0053	0.1599	0.305	0.0479	0.821	0.843	0.0255
SALS 45	ALS	1.03	0.19	148.14	37.92	0.74	0.31	0.34	892.32	1547.62	564.94	607.701	0.0041	0.1526	0.277	0.0209	0.049	0.142	0.0003
C9-ALS7	ALS	0.85	0.38	191.47	43.1	3.92	0.28	0.66	79.69	170.69	211.67	444.277	0.0114	0.1395	0.316	0.0442	0.080	0.261	0.0012
SALS 39	ALS	0.83	4.58	150.18	14.58	1.53	0.37	0.61	474.87	5287.21	241.93	422.481	0.0084	0.1879	0.185	0.0113	0.042	0.156	0.0003
SALS 13	ALS	0.3	2.15	298.18	40.56	1.81	0.61	0.12	4078.48	5401.61	439.78	573.283	0.0042	0.0246	0.563	0.0123	0.088	0.238	0.0000
SALS 38	ALS	0.17	1.03	143.21	22.84	1.38	0.34	0.37	2191.82	1904.31	188.96	332.408	0.004	0.1117	0.256	0.0151	0.195	0.299	0.0004
SALS 35	ALS	0.66	0.56	177.43	26.2	0.75	0.44	0.24	2068.79	4781.97	132.02	613.223	0.0104	0.0533	0.176	0.0143	0.025	0.308	0.0031
SALS 16	ALS	0.64	2.63	166.25	18.19	0.85	0.43	0.55	8991.93	18552.6	215.86	436.195	0.0072	0.1168	0.269	0.0146	0.097	0.177	0.0005

CON1	CON	0.8	9.45	433.07	43.25	2.32	0.53	0.94	2891.36	3997.75	211.407	638.513	0.0087	0.16969	0.358	0.0062	0.079	0.315	0.0070
CON2	CON	1.33	6.31	886.37	40.21	3.12	1.04	0.18	8187.01	29116.1	319.975	349.277	0.01673	0.36734	0.462	0.0138	0.064	0.669	0.0160
CON3	CON	0.67	22.31	86.23	77.73	0.38	1.49	0.09	10545.7	34834	361.683	631.919	0.01553	0.16169	0.718	0.0113	0.323	1.895	0.0197
CON4	CON	0.8	25.89	95.4	86.18	1.78	1.66	1.72	11597.8	8958.27	499.791	912.829	0.03414	0.40563	0.818	0.0075	0.099	1.622	0.0171
CON5	CON	0.17	7.24	316.84	47.65	2.25	0.32	1.37	1218.25	12666.4	171.675	379.999	0.01965	0.28011	0.713	0.0204	0.037	0.120	0.0090
CON6	CON	12.81	11	3364.45	76.12	2.9	1.55	3.19	3392.34	16498.6	915.015	2102.99	0.03302	0.65184	0.790	0.0312	0.112	0.461	0.0048
CON7	CON	31.76	38.82	911.32	180.79	1.32	1.8	0.84	2653.28	12655	1240.45	2070.88	0.01844	0.34645	0.954	0.0126	0.043	0.536	0.0171
CON8	CON	16.98	53.06	1339.44	164.2	13.63	1.62	2.5	2646.58	12027.3	360.44	2451.54	0.05475	0.12916	0.991	0.0546	0.310	1.996	0.0166
CON11	CON	9.88	201.93	1866.93	61.12	1.71	1.57	1.54	2659.1	8017.27	973.044	1763.28	0.02635	0.54463	1.336	0.0128	0.052	0.697	0.0097
CON25	CON	46.14	7.93	132.99	259.5	1.19	1.77	1.05	2589.71	4624.29	457.759	1110.4	0.00626	0.12105	0.419	0.0078	0.008	0.193	0.0193
CON24	CON	0.53	3.09	-82.89	54.44	0.28	3.07	1.42	2599.02	7470.76	153.709	711.581	0.00302	0.02891	0.086	0.0397	0.098	0.410	0.0315
CON26	CON	2.96	23.79	302.56	94.78	3.49	1.64	1.08	2595.98	4729.82	369.38	1348.88	0.01264	0.22386	0.710	0.0529	1.765	0.743	0.0239
CON28	CON	23.35	15.87	1192.28	114.95	2.33	1.68	1.49	2708.2	13035.2	827.087	1695.89	0.06951	0.52029	1.348	0.0249	0.449	0.730	0.0237
CON29	CON	48.51	24.06	2413.9	136.52	1.71	1.87	0.5	2646.8	12997.9	747.758	1514.43	0.01338	0.62633	0.895	0.0205	0.036	0.669	0.0151
CON30	CON	20.47	25.38	1329.58	96.21	2.98	1.6	2.04	3298.75	14934	819.553	2327.04	0.0471	0.45546	1.218	0.0498	0.631	1.635	0.0168
CON27	CON	11.86	9.54	1624.39	69.13	4.84	1.34	0.33	2663.18	14147.9	602.206	1463.2	0.02595	0.8004	1.389	0.0551	0.383	2.796	0.0152
CON33	CON	1.23	7.4	262.2	40.69	1.19	0.65	0.11	12043.9	19758.2	180.103	798.781	0.01031	0.17823	0.489	0.0140	0.168	0.202	0.0009
CON35	CON	0.54	2.25	54.42	34.04	0.94	0.49	0.39	758.371	3727.07	227.468	729.935	0.00632	0.10853	0.166	0.0135	0.023	0.092	0.0170
CON34	CON	3.27	2.6	557.48	29.71	2.8	0.27	0.57	10801.3	17286.5	220.285	633.435	0.01199	0.2256	0.172	0.0277	0.192	0.300	0.0108
CON31	CON	0.12	8.33	180.98	39.06	1.39	0.3	0.2	98.6542	207.969	638.387	629.128	0.00807	0.10681	0.203	0.0161	0.270	0.209	0.0041
CON32	CON	1.83	5.86	172.6	38.3	0.74	0.57	0.39	1336.35	3033.47	334.164	670.714	0.01201	0.15422	0.387	0.0115	0.028	0.415	0.0026
CON23	CON	1.99	6.37	570.17	41.66	0.84	0.55	0.97	4083.13	3131.38	121.891	416.699	0.00646	0.18053	0.252	0.0115	0.038	0.282	0.0006

Table S3. Patient demographics table

	Diagnosis	Age	Gender	Disease duration (mo)	Site of Disease Onset	Analysis used	Notes/Cause of Death
CON1	CON	41	M			RNA-seq MSD	Adenocarcinoma of pancreas; metastatis to diaphragm/lymph nodes
CON2	CON	60	M			RNA-seq MSD	Adenocarcinoma of pancreas; metastatic to liver/diaphragm/pleura/lymph
CON3	CON	50	F			RNA-seq MSD	Metastatic breast cancer to liver, lungs, spleen, lymphnodes, bones.
CON4	CON	72	M			RNA-seq MSD	Cardiorespiratory failure; Pneumonia, acute renal failure
CON5	CON	59	M			RNA-seq MSD	Hypertension, Diabetes Type 2, Transient Ischemic attacks.
CON6	CON	82	F			RNA-seq MSD	Unaffected Control;Diabetes Mellitus
CON7	CON	79	M			RNA-seq MSD	COPD / Peripheral vascular disease
CON8	CON	69	F			RNA-seq MSD	Bacterial endocarditis, atherosclerosi, minor senile changes
CON9	CON	61	M			RNA-seq	Pancreatic adenocarcinoma, liver metastasis
CON10	CON	67	F			RNA-seq	Renal failure; hyperthyroidism
CON11	CON	79	M			RNA-seq MSD	Cardiac infarct; prostatic adenocarcinoma
CON12	CON	68	M			IHC	Unknown
CON13	CON	54	F			IHC	Unknown
CON14	CON	52	M			IHC	Unknown
CON15	CON	55	M			IHC	Unknown
CON16	CON	54	F			IHC	Unknown
CON17	CON	58	M			IHC	Unknown
CON18	CON	50	M			IHC	Unknown
CON19	CON	55	M			IHC	Unknown
CON20	CON	48	M			IHC	Unknown
CON21	CON	72	M			IHC	Unknown
CON22	CON	58	F			IHC	Unknown
CON23	CON	53	M			IHC MSD	Unknown
CON24	CON	74	M			MSD	Neurofibrillary degeneration, Braak stage II
CON25	CON	66	M			MSD	Argyrophilic grain disease, mild
CON26	CON	64	M			MSD	Neurofibrillary degeneration, Braak stage II
CON27	CON	80	M			MSD	Pancratic cancer
CON28	CON	83	F			MSD	Breast cancer/uterine cancer/colon cancer
CON29	CON	61	M			MSD	Normal
CON30	CON	70	M			MSD	Esophageal cancer
CON31	CON	63	F			MSD	Polycystic kidney disease, hemorrhagic shock, cardiac arrest
CON32	CON	51	M			MSD	End stage renal failure, kidney transplant, cardiac arrest
CON33	CON	70	M			MSD	Metastatic cecal carcinoma, multisystem organ failure
CON34	CON	68	M			MSD	End stage renal disease, diabetes, cardiac Arrest

CON35	CON	80	M				IHC	MSD	Unknown
C9-ALS1	C9-ALS	61	F	17		RNA-seq	IHC	MSD	
C9-ALS2	C9-ALS	72	M	42		RNA-seq	IHC	MSD	
C9-ALS3	C9-ALS	61	F	11		RNA-seq		MSD	
C9-ALS4	C9-ALS	51	F	60	bulbar		IHC	MSD	
C9-ALS5	C9-ALS-FTD	74	M	84	Legs		IHC		
C9-ALS 6	C9-ALS						IHC		
C9-ALS7	C9-ALS	61	F	31	limb		IHC	MSD	
C9-ALS8	C9-ALS	68	F	55	limb		IHC	MSD	
C9-ALS9	C9-ALS-FTD	62	M	20			IHC	MSD	
fALS 1	ALS (SOD1 N139K	50	M	74	leg			MSD	
SALS1	SALS	61	F	47	limb	RNA-seq	IHC	MSD	
SALS2	SALS	63	F	24	limb	RNA-seq	IHC	MSD	
SALS3	SALS	68	F	96	limb	RNA-seq	IHC	MSD	
SALS4	SALS	67	M	35	limb	RNA-seq	IHC	MSD	
SALS5	SALS	69	M	21		RNA-seq		MSD	
SALS6	SALS	85	M	170	limb	RNA-seq	IHC	MSD	
SALS7	SALS	74	M	47		RNA-seq	IHC	MSD	
SALS8	SALS	74	M			RNA-seq	IHC	MSD	
SALS9	SALS	60	F	32	limb	RNA-seq	IHC	MSD	
SALS10	SALS	39	M	46		RNA-seq	IHC	MSD	
SALS11	SALS	78	F	36		RNA-seq	IHC	MSD	
SALS12	SALS	81	F	20	bulbar		IHC		
SALS13	SALS	44	M	57	limb		IHC	MSD	**CTE
SALS14	SALS	54	F				IHC		
SALS15	SALS	54	M				IHC		
SALS16	SALS	79	F	19	bulbar		IHC		
SALS17	SALS	63	F	37	bulbar		IHC	MSD	
SALS18	SALS	71	F	25	bulbar		IHC		
SALS19	SALS	63	F				IHC		AD pathology
SALS20	SALS	65	M				IHC		
SALS21	SALS	69	M				IHC		
SALS22	SALS	59	M				IHC		
SALS23	SALS	53	M		limb		IHC	MSD	
SALS24	SALS	68	M	13	Arms		IHC	MSD	
SALS 25	SALS	54	F	37	limb		IHC	MSD	
SALS 26	SALS	58	F	138	limb		IHC	MSD	
SALS 27	SALS	65	F					MSD	

SALS 28	SALS	67	M				MSD	
SALS 29	SALS	70	F				MSD	
SALS 30	SALS	69	M				MSD	
SALS 31	SALS	65	F				MSD	
SALS 32	SALS	68	F	15	limb	IHC	MSD	
SALS 33	SALS	72	F	28	bulbar	IHC	MSD	
SALS 34	SALS	75	M	56	limb	IHC	MSD	
SALS 35	SALS	69	M	23		IHC	MSD	
SALS 36	SALS	61	M	94			MSD	
SALS 37	SALS	66	F	48			MSD	
SALS 38	SALS	55	M	32			MSD	
SALS 39	SALS	56	M	130			MSD	
SALS 40	SALS	51	M	54			MSD	
SALS 41	SALS	68	F	6-9mon			MSD	
SALS 42	SALS	59	M	108			MSD	
SALS 43	SALS	71	M	70		IHC	MSD	**Stroke+meningitis
SALS 44	SALS	61	F	30		IHC	MSD	**Mental delay
SALS 45	SALS	62	F	63		IHC	MSD	
SALS 46	SALS	40	M			IHC	MSD	
SALS 47	ALS-FTD	73	M	19		IHC	MSD	*TDP Pathology

CON 2 and 3

* excluded from RNA-seq analysis

Tabe S4. Choroid plexus samples used for RNA-seq

	Diagnosis	Age	Gender	RIN
CON1	CON	41	M	3.8
CON2 *	CON	60	M	5.3
CON3 *	CON	50	F	2.1
CON4	CON	72	M	4.5
CON5	CON	59	M	7.7
CON6	CON	82	F	3.6
CON7	CON	79	M	4.9
CON8	CON	69	F	6
CON9	CON	61	M	7.2
CON10	CON	67	F	6.1
CON11	CON	79	M	4.9
C9-ALS1	C9-ALS	61	F	8
C9-ALS2	C9-ALS	72	M	6.3
C9-ALS3	C9-ALS	61	F	7
SALS1	SALS	61	F	8.3
SALS2	SALS	63	F	7.3
SALS3	SALS	68	F	8.5
SALS4	SALS	67	M	7
SALS5	SALS	69	M	4.4
SALS6	SALS	85	M	6.7
SALS7	SALS	74	M	5.8
SALS8	SALS	74	M	8.1
SALS9	SALS	60	F	6.9
SALS10	SALS	39	M	7.3
SALS11	SALS	78	F	5.8

* excluded from RNA-seq analysis

Supplemental Table 5: List of Antibodies used

Antibody	Company	Catalog number
CD3	Abcam	ab16669
UEA1 Lectin	Vector labs	B1065
CD31	Abcam	ab28364
CD62P/P-selectin	Abcam	ab6632
CRP	Proteintech	66250-1-Ig
Fibrinogen	Abcam	ab34269
MERTK	Abcam	ab52968
ZO1	Novus	NBP1-85046
PDGFRb	R&D Systems	AF385
CD13/ANPEP	Proteintech	14553-1
vWF	Abcam	ab6994
E-cadherin	Proteintech	20874-1-AP
Occludin	Novus	NBP1-87402
Claudin 3	Novus	NBP1-35668
Claudin 5	Thermo Fisher	35-2500
phospho TDP-43 (409/410)	Millipore	MABN14
